# A Vernalization Response in a Winter Safflower (*Carthamus tinctorius*) Involves the Upregulation of Homologs of *FT*, *FUL*, and *MAF*

**DOI:** 10.3389/fpls.2021.639014

**Published:** 2021-03-30

**Authors:** Darren P. Cullerne, Siri Fjellheim, Andrew Spriggs, Andrew L. Eamens, Ben Trevaskis, Craig C. Wood

**Affiliations:** ^1^Agriculture and Food, Commonwealth Scientific and Industrial Research Organisation, Canberra, ACT, Australia; ^2^Department of Plant Sciences, Norwegian University of Life Sciences, Ås, Norway; ^3^School of Environmental and Life Sciences, The University of Newcastle, Callaghan, NSW, Australia

**Keywords:** safflower, vernalization, genome, PacBio, flowering time

## Abstract

Safflower (*Carthamus tinctorius*) is a member of the Asteraceae family that is grown in temperate climates as an oil seed crop. Most commercially grown safflower varieties can be sown in late winter or early spring and flower rapidly in the absence of overwintering. There are winter-hardy safflower accessions that can be sown in autumn and survive over-wintering. Here, we show that a winter-hardy safflower possesses a vernalization response, whereby flowering is accelerated by exposing germinating seeds to prolonged cold. The impact of vernalization was quantitative, such that increasing the duration of cold treatment accelerated flowering to a greater extent, until the response was saturated after 2 weeks exposure to low-temperatures. To investigate the molecular-basis of the vernalization-response in safflower, transcriptome activity was compared and contrasted between vernalized versus non-vernalized plants, in both ‘winter hardy’ and ‘spring’ cultivars. These genome-wide expression analyses identified a small set of transcripts that are both differentially expressed following vernalization and that also have different expression levels in the spring versus winter safflowers. Four of these transcripts were quantitatively induced by vernalization in a winter hardy safflower but show high basal levels in spring safflower. Phylogenetic analyses confidently assigned that the nucleotide sequences of the four differentially expressed transcripts are related to *FLOWERING LOCUS T (FT)*, *FRUITFUL (FUL)*, and two genes within the *MADS-like* clade genes. Gene models were built for each of these sequences by assembling an improved safflower reference genome using PacBio-based long-read sequencing, covering 85% of the genome, with N50 at 594,000 bp in 3000 contigs. Possible evolutionary relationships between the vernalization response of safflower and those of other plants are discussed.

## Introduction

Vernalization is the acceleration of flowering by exposure to the prolonged cold of winter (Chouard, 1960). In temperate regions, vernalization coordinates the plant life cycle with the changing seasons by delaying flowering before winter, thereby avoiding freezing/chilling damage to reproductive organs. By promoting rapid flowering in spring, vernalization also allows some plants to flower before the onset of heat and dry conditions in summer. Vernalization is widespread in flowering plants, occurring in both dicots and monocots. Many crops cultivated in temperate regions exhibit vernalization-induced flowering, including wheat (*Triticum aestivum*), oilseed rape (*Brassica napus*), pea (*Pisum sativum*), and sugar beet (*Beta vulgaris*) (Chouard, 1960).

The molecular basis of vernalization-induced flowering was first resolved in the model plant Arabidopsis (*Arabidopsis thaliana*). The vernalization response of Arabidopsis is mediated by the *FLOWERING LOCUS C (FLC)* gene, which encodes a MADS (MCM1/AGAMOUS/DEFICIENS/SRF) box transcription factor protein (Michaels and Amasino, 1999; Sheldon et al., 1999). *FLC* delays flowering before winter by repressing transcription of genes that would otherwise promote flowering, including *FLOWERING LOCUS T (FT)*, which accelerates flowering in long days (Michaels et al., 2005; Helliwell et al., 2006). Transcription of *FLC* is repressed by vernalization (Michaels and Amasino, 1999; Sheldon et al., 1999). This repression is retained post-vernalization through the action of protein complexes that change the state of chromatin at the *FLC* locus (Bastow et al., 2004; Schubert et al., 2006; Wood et al., 2006; Finnegan and Dennis, 2007). Thus, plants retain a molecular memory of winter and flower rapidly when exposed to normal growth temperatures and long days after vernalization. *FRIGIDA (FRI)* is required for high levels of *FLC* expression prior to vernalization and loss of *FRI* gene function leads to early flowering without the normal requirement for vernalization (Johanson et al., 2000). *FLC*-like genes mediate vernalization-induced flowering in other *Brassicaceae*, including oilseed rape (Tadege et al., 2001; Irwin et al., 2016; O’Neill et al., 2019; Tudor et al., 2020; Yin et al., 2020). As outlined below, the molecular basis of vernalization has been resolved to varying extents in plants outside the *Brassicaceae*.

In wheat, *VERNALIZATION 2 (VRN2)* encodes a zinc-finger CCT (CONTANS, CONSTANS-LIKE, TOC1) domain protein that blocks long-day induction of *FT* (*FT1* in wheat) and thereby delays flowering before winter (Yan et al., 2004). Vernalization activates transcription of *VRN1*, a promoter of flowering related to the *APETALA1* (*AP1*) and *FRUITFUL* (*FUL*) MADS box genes of Arabidopsis (Danyluk et al., 2003; Trevaskis et al., 2003; Yan et al., 2003). Activation of *VRN1* is maintained post-vernalization and this is associated with changes in the state of chromatin at the *VRN1* locus (Oliver et al., 2009). Elevated expression of *VRN1* then down-regulates *VRN2* and a second repressor of flowering, *ODDSOC2 (OS2)*, a MADS box gene that appears to have evolved from the *FLC*-clade of MADS box gene family (Greenup et al., 2011; Ruelens et al., 2013). The VRN1 protein binds directly to sites in the *VRN2*, *OS2*, and *FT1* genes, suggesting that the VRN1 protein triggers vernalization-induced flowering by directly regulating both repressors and activators of flowering (Deng et al., 2015). Naturally occurring mutations in the *VRN1* gene that activate expression of this gene without prolonged exposure to cold are the main driver of the reduced vernalization requirement in cultivated “spring” wheats (Fu et al., 2005). In barley (*Hordeum vulgare*), loss-of-function mutations in *VRN2* are another driver of reduced vernalization requirement, in addition to active alleles of *VRN1* (Yan et al., 2004). There are also examples of mutations that activate *FT1* expression and thereby bypass the normal need for vernalization-induced flowering of wheat and barley (Yan et al., 2006).

There are several examples of vernalization-responsive species amongst the temperate legumes, including chickpea, pea, narrow leaf lupin (*Lupinus angustifolius*), and *Medicago* species. In the model legume *Medicago truncatula*, the *FT*-like gene *FTa1* is upregulated by vernalization and by long days. Mutations that disrupt *FTa1* function delay flowering of vernalized plants (Laurie et al., 2011). Conversely, genetic activation of *FTa1* in transgenic plants or by retroelement insertion leads to early flowering without vernalization (Laurie et al., 2011; Jaudal et al., 2013; Yeoh et al., 2013). Taken together these findings suggest that *FTa1* plays a key role in mediating vernalization-induced flowering of legumes. The *FTa1* gene is not transcriptionally activated by vernalization *per se*, suggesting that a different mechanism mediates the actual response to prolonged cold in legumes (Laurie et al., 2011).

**FIGURE 1:**
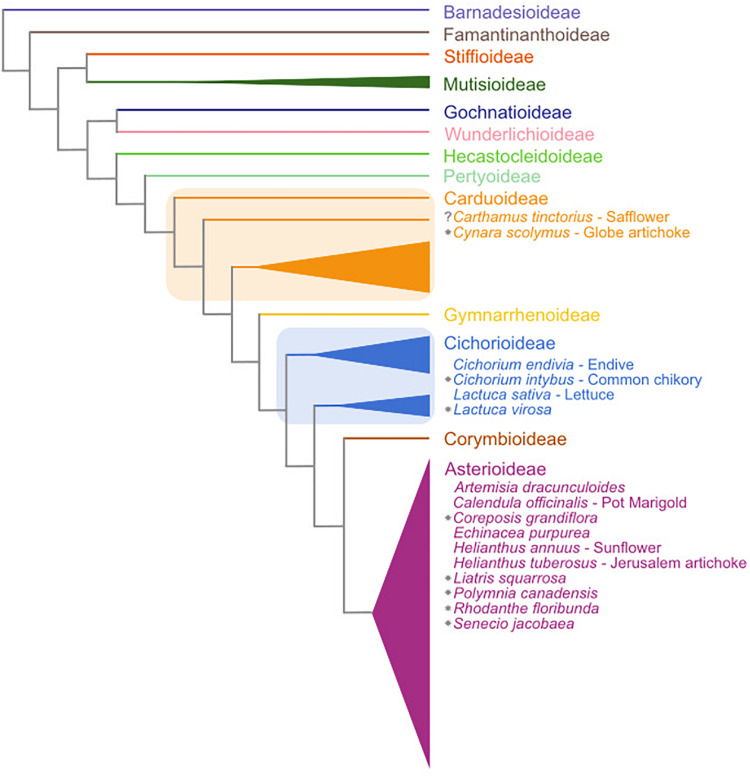
Schematic overview of the subfamilies of the Asteraceae family based on Mandel et al. (2019). Central Asteraceae crop species are listed in their respective subfamilies. Gray stars indicate species that have a documented vernalization response in flowering. Subfamily assignments are based on Fu et al. (2016) and Mandel et al. (2019). Note that two of the subfamilies (Carduoideae and Cichorioideae) are paraphyletic and are thus marked with boxes.

The Asteraceae family is the largest amongst the flowering plants, comprising 25000–35000 species that represent approximately 10% of all angiosperm species (Mandel et al., 2019). The Asteraceae contain several economically important species, examples being crops like lettuce (*Lactuca sativa*) and sunflower (*Helianthus annuus*). Many Asteraceae are adapted to temperate climates and vernalization-induced flowering has been described in several species (Harwood and Markaria, 1968; Baskin and Baskin, 1989; Prins et al., 1990; Bender et al., 2002; Niu et al., 2002; Ha and Johnston, 2013; Perilleux et al., 2013) (see Figure 1). The molecular basis for vernalization-induced flowering in these plants is not well understood.

Safflower (*Carthamus tinctorius* L.) is a member of the Asteraceae family that is cultivated to produce seed oils, traditional medicines and dyes (Zohary et al., 2012). Genetically modified safflower has also been used to produce oils with distinct properties not normally found or easily produced in nature (Nykiforuk et al., 2012; Wood et al., 2018). The commercial safflower cultivars grown in Australia, North America and Mexico are sown in late winter or early spring and flower rapidly without over-wintering. Safflower is considered a long-day plant (Dole, 2015) and although “spring” cultivars dominate global safflower production there are reports that some accessions can be sown in autumn and survive winter conditions that are too extreme for spring safflower (Ghanavati and Knowles, 1977; Yazdisamadi and Zali, 1979). These same winter hardy cultivars have delayed flowering and reduced seed set when sown in spring. This was attributed to a lack of cold stimulation during the growth period (Yazdisamadi and Zali, 1979), but a vernalization response has not yet been demonstrated.

High-quality genomes are increasingly important resources for plant breeders, with accurate DNA sequencing and long contigs being noteworthy features. Safflower has a 1.35 Gb genome distributed on 12 chromosomes. While there are existing genomic resources available for safflower (Bowers et al., 2016), these have been generated with so called ‘short read’ technologies (100 bp reads; Bentley et al., 2008). To date, the most current safflower assembly is fragmented (57,000 contigs with an average contig length ∼2000 bp) and is estimated to cover less than 70% of the genome. The development of ‘long read’ technologies (>10,000 bp reads, Eid et al., 2009) addresses these shortcomings by enabling the construction of assemblies with far longer contigs, and potentially a significantly improved physical assembly.

Here, using controlled condition experiments, we show that a winter hardy safflower accession is vernalization responsive. Using transcriptome sequencing to compare gene-expression patterns in “spring” versus “winter” safflower varieties we then identify a small set of genes that potentially mediate vernalization-induced flowering in safflower. Gene models of these transcripts, including their long intronic sequences, were generated using a significantly improved genome assembly based on PacBio technologies. The evolutionary implications of these findings are discussed.

## Materials and Methods

### Plant Materials and Growth Conditions

Two safflower varieties were used in this study: ‘winter hardy’ C311, known to survive extended cold periods (including overwintering under snow) and ‘spring’ S317, which is a widely grown commercial cultivar (Wood et al., 2018). C311 was sourced from US Department of Agriculture Genetic Resources Information Network (PlantID WSRC03, Johnson and Dajue, 2008). Prior to more detailed analysis of flowering time in growth cabinets, S317 and C311 were cultivated in parallel in a small experimental plot at CSIRO Black Mountain site (alluvial soil) in late spring. This plot demonstrated that C311 was significantly later to flower than S317 in the absence of a defined cold period (see Supplementary Figure 1).

For detailed vernalization experiments safflower seeds were treated as described below. Specifically, seeds were imbibed overnight by hydrating in aerated distilled water at room temperature. After 24 h, seeds were visually inspected for a cracking of the seed coat, and any seed not cracked was discarded. Imbibed seeds were placed onto moistened filter paper disks within plastic petri dishes, covered in foil and the temperature maintained at 4°C for varying durations (vernalization). Similarly, vernalization at different temperatures was performed by placing imbibed seeds into 15 mL falcon tubes and incubating in Echotherm programmable temperature blocks (Torrey Pines Scientific Instruments, CA, United States), set to desired temperatures and each experiment used a 20-day vernalization period that had been found to be saturating in preliminary tests. Following all vernalization treatments, germinated seeds were transferred to 20 cm pots with soil and buried at 2 cm depth. Soil comprised of 70:30% soil perlite mixture, with a small quantity of slow release fertilizer (Osmocote) and liquid fertilizer (Aquasol) applied occasionally throughout growth. In all controlled growth chambers plants were grown in simulated long-day conditions (26°C, 16 h light at approximately 450 μM.m^–2^s^–1^; Conviron, Canada) for recording of flowering time. Flowering time was recorded as the time when the floret on the main stem had emerged. Vernalization days were calculated, using a method outlined previously (Baloch et al., 2003) and the days to heading were adjusted accordingly. The relationship between length of vernalization exposure at 4°C and heading date for each of the two cultivars were tested with regressions. Differences in heading date between plants treated with different vernalization temperatures over a 28-day duration were tested with an ANOVA, followed by a Tukey Honestly Significant Difference (HSD) test performed in the R stats package (R Core Team, 2016).

### Genetic Analysis of Growth Habit

Crossing was performed as outlined previously (Mundel and Bergman, 2009). Then, the progeny were grown in warm, long-day conditions (see above for conditions) and the timing of bolting (when stem elongation begins) was used to differentiate between winter and spring growth habit; bolting before 4 weeks indicated spring growth habit, whereas bolting taking longer than 4 weeks indicated a winter growth habit. All F_1_ seeds were grown to maturity, then the F_2_ progeny were grown and scored in a similar manner.

### Assembling an Improved Safflower Reference Genome

Nuclear genomic DNA from S317 was isolated in a method as previously described (Naim et al., 2012). Genomic DNA was sequenced using both Illumina (Bentley et al., 2008) and PacBio (Eid et al., 2009) chemistries. Illumina-based methods used the HiSeq2000 instrument at the Australian Genome Research Facility, Melbourne, and generated Paired End (PE) reads (100 bp with an insert length of 180 bp) and Mate Pair (MP) reads (36 bp read length with an insert length of 10 Kbp). PE reads were first processed for quality by visual examination with FastQC (Andrews et al., 2012) then using BioKanga “filter” (v3.1.1) to remove any reads that: were duplicates; that did not overlap by at least 50% with another read; or contained more than a single ambiguous base. A *de novo* assembly was constructed with the PE reads using BioKanga “assemb” protocol, then repeated combining the *de novo* PE assembly and MP reads. The second *de novo* assembly was scaffolded twice with BioKanga “scaffold,” first using a fragment size of 180 bp, then again with 10 Kbp. The second scaffolded *de novo* assembly was further scaffolded with SCUBAT (Elsworth and Weitemier, 2013) using a *de novo* reference transcriptome for S317, as constructed previously (Wood et al., 2018). PacBio-based reads were acquired from a RSII sequencer (Version 6 chemistry) at the Queensland University of Technology Diamantina Institute, Brisbane. A completely independent genome assembly using the PacBio reads was constructed using Canu software (v1.5, Koren et al., 2017), requiring a minimum read length of 10 Kbp and an estimated genome size of 1.4 Gbp. The overall quality and coverage of the *de novo* assemblies was assessed using BUSCO (v3.0.2) (Simao et al., 2015) using the core gene set downloaded from the “embryophyta_odb9” reference database.

### Transcriptome Analysis

Seeds were germinated and vernalized in the dark for 5, 10, 15, or 20 days at 4°C as described above, then sown in pots in long day conditions (as described above) for 1 week before RNA extraction. By this stage plants had emerged from the soil, cotyledons were fully expanded and two true leaves were present. Non-vernalized controls were germinated in a similar manner but shifted directly to long-day growth conditions and grown in for a week. At approximately 3 h after dawn the collar (transition between root and stem) of each plant was located and a location 2 mm below the collar was cut and all tissue above that location was harvested for further analysis. Tissue samples were then frozen in liquid nitrogen and kept at −80°C until further processing. Total RNA was extracted using PureLink reagent (cat#: 12322-012, Life Technologies) following the manufacturer’s instructions. RNA, enriched in poly-adenylated RNA (Epicenter Technologies, United States), was sequenced at the Australian Genome Research Facility (AGRF) using an Illumina HiSeq2000 sequencer, using 100 bp paired-end reads. Reads were adapter filtered and aligned against a *de novo* reference transcriptome for S317 as constructed previously (Wood et al., 2018) using BioKanga ‘Align’ (v3.8.1)^1^ with default settings.

Aligned read counts were normalized and analyzed using DESeq2 (v1.6.3) (Love et al., 2014), using a model that identifies transcripts that are differentially expressed between spring and winter safflower cultivars (summarized as non-vernalized S317 vs. non-vernalized C311, or nvS317:nvC311) and as the duration of exposure to vernalization conditions increased (non-vernalized C311 vs. vernalized C311, or nvC311:vC311). Transcripts were identified that were significantly differentially expressed (adjusted *p*-value < 0.05) between winter and spring safflower and across all time points. Initial visualizations of the intersections between groups of differentially expressed genes we used the UpSetR plotting package in R.

Additionally, a *de novo* transcriptome assembly was created for the winter safflower accession, C311, using the BioKanga ‘Assemb’ and ‘Scaffold’ software (v3.5.3 – see text footnote 1) with the default parameters (hereby referred to as the “winter safflower transcriptome” and using the naming nomenclature “CarTin_tx_WSRC03_Scaff<# > _ < #>”). This assembly of the C311 transcriptome was used primarily to examine variation in transcripts homologs between winter and spring safflower and to compare against genes from other plant species.

After the identification of transcripts that were differentially expressed in winter and spring varieties and during vernalization treatments, a simple BLAST search (NCBI default parameters, Altschul et al., 1997) was used to conduct an initial identification of genes putatively involved in vernalization in safflower.

### Real-Time PCR Analysis of Candidate Genes

Primers were generated using Oligo Explorer^2^ (v1.1.2) and with Netprimer^3^ (v3). Primers were also developed for *CtActin* as a normalizing gene in the analysis (Czechowski et al., 2005). Primers were designed from the 3′ end of the transcript, with at least 18nt and a predicted melting temperature (*T*_*m*_) above 62°C (Sigma-Aldrich Inc., Sydney, NSW, Australia; Supplementary Table 1) with a final amplicon length in the range of 100–200 bp. Amplicons were cloned and sequenced to confirm their target sequence.

Total RNA samples from each time point in the time course treated with RQ1 DNase (M6101, Promega) with Maxima Reverse Transcriptase (Thermo Fisher Scientific) for first strand cDNA synthesis. Four biological samples were used and each of these were split to generate four technical replicates for real-time PCR analysis. Each 20 μL reaction contained 2 μL reaction buffer, 1.4 μL of 50 mM MgCl_2_, 1 μL Fast SYBR Green (Thermo Fisher Scientific), 0.5 μL of 10 μM forward and reverse primer, 0.8 μL of 10 mM dNTPs and 0.1 μL Platinum Taq Polymerase (Thermo Fisher Scientific) and 20 ng first strand cDNA template. PCR amplification was performed on a Rotor Gene Q (Qiagen). Thermal cycling conditions were 95°C for 5 min followed by forty-five cycles of 95°C for 20 s, 59°C for 20 s and 72°C for 20 s. This was followed by a melt curve analysis consisting of cooling samples to 50°C before increasing the temperature to 99°C in 1°C increments, and by holding for 5 s at each increment.

### Phylogenetic Analysis of Candidate Transcripts

We used phylogenetics to conduct a more thorough characterization of transcripts, using well-characterized gene families from Arabidopsis as a reference. Genes putatively related to MADS-box transcription factors and FT-like were compared against the serum response factor (SFR)-type transcription factor gene family (pfam00319) and the phosphatidylethanolamine-binding protein (PBP) gene family (pfam01161), respectively, as downloaded from the pfam database (El-Gebali et al., 2019). Sequences from the sunflower (*Helianthus annuus)* genome were used as a reference for the Asteraceae family. Pfam reference numbers were matched to UniProt accessions [The UniProt Consortium 2019 (Morgat et al., 2020)] and duplicates were removed so that each unique genome locus was represented only by one sequence. The sequences from Arabidopsis, safflower and differentially expressed safflower transcripts were aligned using MUSCLE (Edgar, 2004) through the EMBL-EBI web service (Madeira et al., 2019). An initial ‘maximum-likelihood’ (ML) phylogenetic analysis of the SFR-type transcription factor gene family (MADS-box) using PHY-ML with default settings, including a LHRt support value assessment, revealed that the safflower transcripts belong to the MIKC-MADS box group of SFR-type genes (Parenicova et al., 2003). Thus, the analysis was repeated but only including the MIKC-MADS box genes and using PHY-ML with default values, except that a bootstrap evaluation with 250 replicates was performed. The ML trees were midpoint rooted. For the PBP (FT) gene family a ML phylogenetic analysis was performed in PHY-ML (Dereeper et al., 2008) using default values, except from validating the phylogeny with 250 replicates in a bootstrap analysis. Two sequences were removed as they did not align to the rest of the sequences and clustered outside the other sequences and were assumed to likely represent falsely annotated sequences (At5g01300 and HannXRQ_Chr10g0291491). Four sunflower sequences were removed from the analysis as they were shorter than 100 amino acids.

To investigate the presence of transcripts more closely related to the Arabidopsis *FLC* than the noteworthy candidate transcripts identified in our experiment, we did a BLAST search with Arabidopsis *FLC* against the transcriptomes of S317. The best hits were included in a phylogeny with sequences from the *MAF-* and *FUL-*clades from the larger MIKC MADS phylogeny following the same setup for analysis as described above. Similarly to investigate the presence of a safflower transcript being closely related to the *Cichorium intybus FLC-like* gene, *CiFL1*, we conducted a BLAST search of the safflower genome, and the closest hit was included in a further phylogenetic analysis as described above.

### Gene Models

The noteworthy transcripts from the *de novo* transcriptome that were identified as candidates for control of vernalization response (see previous section) were aligned against the *de novo* genomic reference using BioKanga ‘Blitz’ to define the structure of the genes. The settings used were a minimum of 40% alignment, a mismatch penalty score of 2 and a maximum over-exploring seed depth of 10,000. Intron/exon boundaries were identified by large alignment gaps when mapping the transcriptomic contigs. Upstream and downstream untranslated regions were identified by transcriptomic alignments before start and after stop codons, respectively.

## Results

### Vernalization-Responsive Flowering of a Winter-Hardy Safflower

**FIGURE 2:**
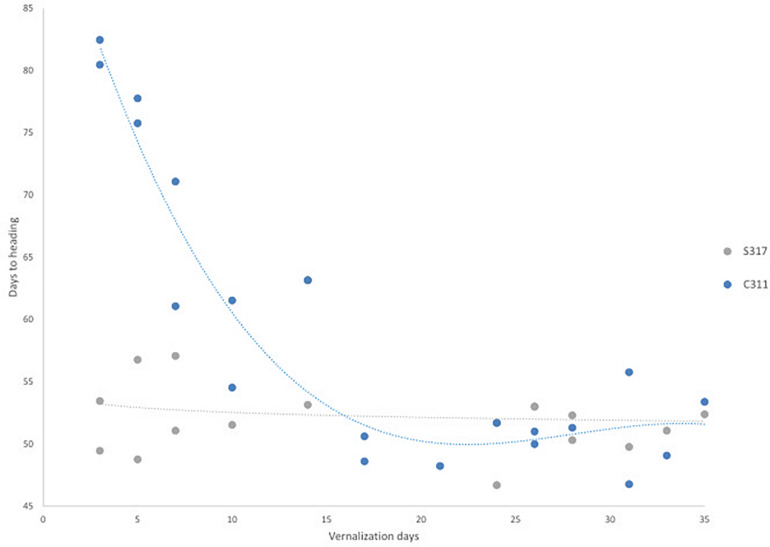
The effect of vernalization on flowering time for S317 and C311. Germinated seeds were exposed to increasing longer durations of a vernalization (4°C) treatment. In C311, there is a steep decrease in the time to flowering as the length of vernalization increases and the relationship between duration of vernalization and days to heading was significant. This slope levels out at approximately 17 days and does not decrease the time to flowering after further exposure to vernalization conditions.

The flowering behavior of a modern spring safflower cultivar (S317) and a winter hardy safflower (C311) were compared in different growth habitats, including growth chambers under long-day conditions, 26°C, 16 h of light at approximately 450 μM.m^–2^s^–1^) and in small field plots (Supplementary Figure 1). Preliminary experiments indicated that in the absence of exposure to cold the two varieties had visibly different growth habits, where C311 was always slower to bolt than S317. More detailed studies were conducted in growth chambers under long-day conditions and S317 bolted rapidly in these growth conditions and produced the first flower after approximately 50 days, whereas C311 was slower to bolt, formed a larger rosette and produced the first flower after 80 days (Figure 2). To test whether the delayed flowering of C311 was due to a vernalization requirement, imbibed seeds were exposed to cold (4°C) for different durations before being placed in long-day conditions, and then the days until the appearance of the first flower recorded. Exposing imbibed seeds of C311 to prolonged cold reduced the number of days required for the first flower to appear from ∼80 days to ∼45–50 days (Figure 2). Simple linear regression showed a significant relationship between the duration of cold exposure and the number of days until the first flower appearance for C311 (*p* < 0.001, slope coefficient −0.889, *R*^2^ 0.662). In contrast, there was no significant relationship between cold treatment and flowering time for the spring safflower S317 (*p* = 0.229, slope coefficient −0.070, *R*^2^ 0.095). The maximal acceleration of flowering of C311 was observed following 14 days of cold pre-treatment of imbibed seeds. In these experiments the seeds of cold treated plants were collected and these progeny seed were re-tested for their response to a cold-treatment and the hastening of flowering time in C311 was confirmed. Overall, these observations are consistent with winter safflower possessing a vernalization requirement. Comparison of vernalization at different temperatures showed that there were significant differences in heading dates between the temperature applied (Supplementary Figure 2; *F* = 14.11, *p* < 0.001). Temperatures between 0°C and 12°C degrees were equally effective for vernalization of safflower, however treatment with 16°C was less effective for hastening heading date. We used a vernalization temperature of 4°C in subsequent experiments and a vernalization treatment of longer than 14 days was considered a saturating response in C311.

### Transcriptome Analysis of Vernalization Response of Safflower

**FIGURE 3:**
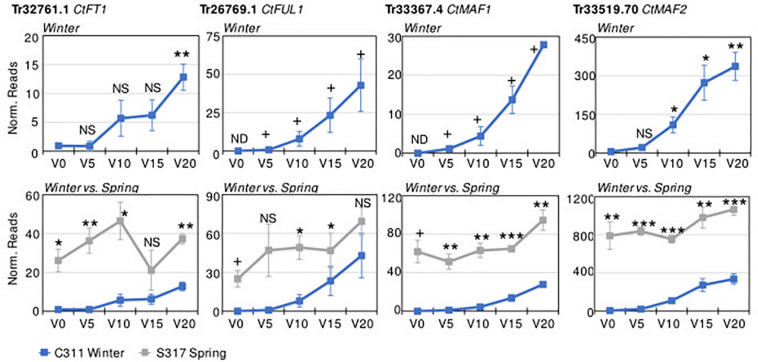
Differential expression of key safflower transcripts with vernalization treatment and genotype. Plotted on the *y*-axes are the average normalized transcript read counts, from three biological replicates for key transcripts from transcriptomes of non-vernalized plants (V0) or plants that had been vernalized for 5, 10, 15, or 20 days. Data are presented for the winter safflower accession (C311, blue line), showing the impact of increasing durations or vernalization pre-treatment (top row). Then, gene expression in the winter type is contrasted with the spring cultivar (S317, gray line). Error bars show standard error. Statistical tests include Student’s *t*-test comparison to the non-vernalized control for time course analysis of expression in the winter safflower (when plotted alone) or comparison between the spring versus winter safflower when genotypes are compared (NS, non-significant, *P < 0.05, **P < 0.01, ***P < 0.001). Expression not detected in some samples (ND), resulting in an absolute or presence/absence contrast (+).

Transcriptome sequencing (RNASeq) was used to identify vernalization-responsive genes in winter safflower. Specifically, transcriptomes were generated from young seedlings of both the spring and winter safflower, grown in long-day conditions from non-vernalized seeds or from seeds exposed to cold for 5, 10, 15, or 20 days. We found that more than 4000 genes were differentially expressed across all of the treatments (Supplementary Figure 3), however, as a subset of these various classes of changes we were specifically interested in genes that were differentially expressed both between the different non-vernalized genotypes (S317 vs. C311) and in response to vernalization (C311 non-vernalized vs. C311 vernalized). This analysis identified 57 transcripts as being significantly differentially expressed according to these combined criteria (Supplementary Figure 3 and Table 1). Of these 57 transcripts, only 14 unique sequences showed greater than two-fold change in expression (i.e., normalized read counts) both between genotypes and in response to vernalization (Table 1). As an additional round of assessment we also inspected the time course of transcript abundance for each of these transcripts to identify those genes that responded to vernalization in a dose-dependent manner, consistent with the response to extended cold treatment. This analysis pipeline found four transcripts (Tr32761.1, Tr26769.1, Tr33367.4, and Tr33519.70) that showed a quantitative response to vernalization, with increasing transcript abundance in seedlings exposed to progressively longer cold treatments, and these four transcripts all had higher expression in the S317 accession relative to C311 (Figure 3). Two other transcripts, Tr870612.1 and Tr636776.1, were induced by vernalization in the spring safflower (Supplementary Figure 4). Another transcript, Tr123834.1, had higher levels in non-vernalized seedlings of the winter safflower relative to the spring type (Supplementary Figure 4). Levels of Tr123834.1 were lower in winter safflower plants vernalized for more than 5 days but showed the opposite response to vernalization in the spring type (Supplementary Figure 4). Other differentially expressed transcripts included Tr69290.1, which was repressed by vernalization in the winter safflower, and Tr31946.1 that showed large differences in transcript levels between the spring and winter safflowers but no similarity to genes of known function (Table 1 and Supplementary Figure 4). The remaining transcripts detected by the differential expression analysis showed no clear overall relationship between read counts, genotype or exposure to vernalization (Supplementary Figure 4).

**TABLE 1:** List of 14 differentially expressed safflower transcripts identified during a vernalization treatment in a winter variety and in a comparison of transcripts differentially expressed in a non-vernalized treatment of the winter and spring variety.

Transcript	Top match putative annotation	Log_2_FC C311V:CS311NV	Log_2_FC S317NV:C311NV
Tr26769.1	MADS FUL-like	>10	>10
Tr33367.4	MADS FLC-like	>10	>10
Tr33519.70	MADS FLC-like	6	7.2
Tr32761.1	FT-like	3.8	4.8
Tr849506.1	Unknown	3.4	6
Tr4835.1	Proteinase inhibitor	1.1	2.4
Tr355653.1	HMG-CoA reductase	1	2.1
Tr32216.1	Zinc finger, RING/FYVE/PHD-type	−1	−1.4
Tr59634.1	Unknown	−1.1	−1.4
Tr18241.1	Unknown	−1.2	−1.3
Tr123834.1	RAN BP2 zinc finger	−1.3	−1.5
Tr69290.1	Methylene-furan-reductase-like	−2.6	−2.1
Tr31946.1	Unknown	−3.4	+5
Tr636776.1	Unknown	−3.4	+1.6

*Log_2_FC_C311V:C311NV, refers to the log_2_ fold-change between a vernalized (V) and a non-vernalized (NV) treatment on a winter variety (C311). Log_2_FC_S317NV:C311NV, refer to the log_2_ fold-change between a transcript in a non-vernalized spring variety (S317) and non-vernalized winter variety (C311). Transcripts were BLASTED to provide a putative annotation. The abundance of the transcripts during a time course of vernalization is graphed in Figure 3 and Supplementary Figure 2.*

We used RT-qPCR to access the abundance levels of both Tr32761.1 and Tr33367.4 in S317 and C311 during a vernalization timecourse. Both transcripts were constitutively expressed in S317, but the abundance of both transcripts was below detection limits at both zero and 5 days vernalization in C311, yet both transcripts became detectable after 10, 15 and 20 days of cold treatment (Supplementary Figure 5). These results based on RT-qPCR are in agreement with conclusions from methods based on RNASeq.

### Sequence Relationships Between Vernalization-Responsive Transcripts of Safflower and Genes From Other Plants

**FIGURE 4:**
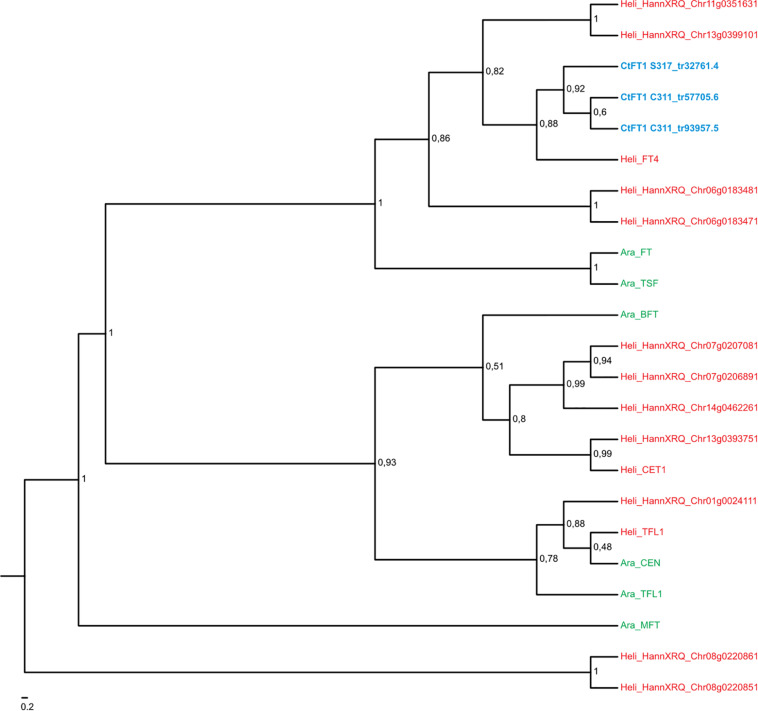
Maximum likelihood phylogeny of the MIKC-MADS gene family in *Arabidopsis thaliana* (in green) and *Helianthus annum* (in red). Transcripts of *Carthamus tinctorius* (in blue) found to be differentially expressed in response to vernalization were included. Bootstrap support values are shown for nodes with higher than 50% support. *CtMAF1* sequences from spring safflower (S317_tr33367.4 and S317_tr33367.5) appear to be isoforms of the same transcript, with variations found in the 3′ end of S317_tr33367.4. Similarly, the *CtMAF1* sequences from the winter safflower transcriptome (C311_tr20021.6 and C311_tr23886.4) also appear to be isoforms of the same transcript, with a 5′ truncation and single codon gap in C311_tr23886.4. Information about the genes can be found in Supplementary Table 2.

Comparisons with DNA databases showed the four transcripts that exhibited quantitative responses to vernalization in C311 (namely Tr32761.1, Tr26769.1, Tr33367.4, and Tr33519.70) are related to genes known to regulate flowering in other plants (Table 1). A simple BLAST search revealed that Tr32761.1 is similar to *FT-like* genes in other plants (Table 1). Phylogenetic analyses further showed that Tr32761.1 grouped with the Arabidopsis *FT* and *TWIN SISTER OF FT* genes in a monophyletic clade with high bootstrap support (bs = 1, Figure 4). Thus, hereafter we refer to Tr32761.1 as the safflower ortholog of the *FT* gene, namely *CtFT1*.

**FIGURE 5:**
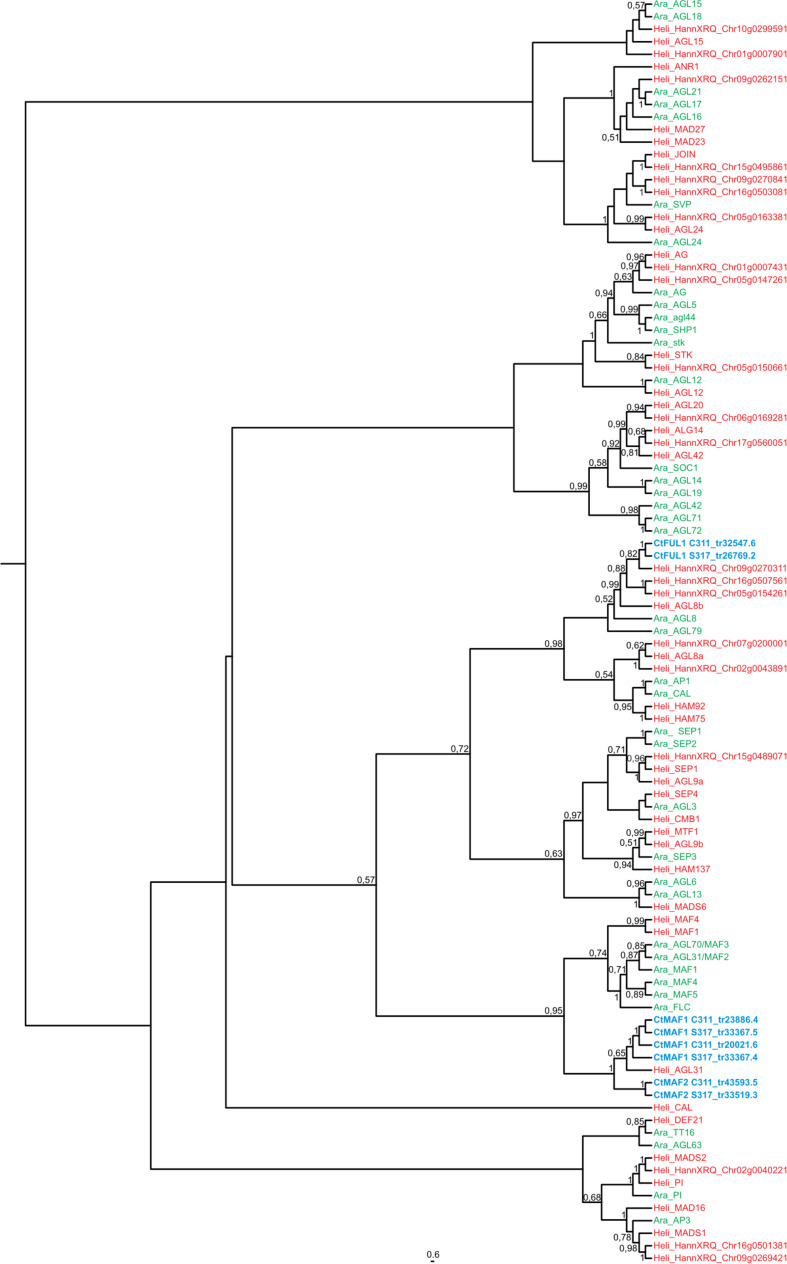
Maximum likelihood phylogeny of the *FT* gene family in *Arabidopsis thaliana* (in green) and *Helianthus annum* (in red). Transcripts of *Carthamus tinctorius* (in green) found to be differentially expressed in response to vernalization were included. Bootstrap support values are shown for nodes with higher than 50% support. Regarding the two transcripts from the winter safflower transcriptomic assembly, namely C311_tr57705.6 and C311_tr93957.5. The former is a transcript containing a truncation at the 3′ end that closely resembles the CtFT1 from the spring safflower transcriptome (S317_tr32761.4). The latter closely resembles C311_tr57705.6 but also contains a 5′ truncation. Information about the genes can be found in Supplementary Table 2.

A BLAST search revealed that the sequence Tr26769.1 sits in the large MADS box gene family, and more similar to *AP1/FUL*-like genes from other plants. More in-depth phylogenetic analyses placed Tr26769.1 in a monophyletic cluster with high bootstrap support (bs = 0.98), together with the Arabidopsis *AP1*, *CAL* and *FUL* genes (Figure 5). Precise relationships between the safflower sequence and the different Arabidopsis MADS box genes within the clade are uncertain because bootstrap support for a key branch node is weak. Nevertheless, the Tr26769.1 is unlikely to be a direct ortholog of the Arabidopsis *AP1* gene, as it was not placed within the well supported (bs = 0.95) clade with *AP1* and *CAL*. Therefore, we refer to Tr26769.1 as a safflower homolog of *FUL*, namely *CtFUL1*.

A BLAST search indicated that Tr33367.4 and Tr33519.70 also showed sequence similarity to *MADS*-box genes, with the strongest similarity to genes annotated as being ‘*FLC*-like.’ Phylogenetic analysis placed the safflower sequences in a clade with the Arabidopsis *FLC* gene family, which includes *FLC* and the *MADS AFFECTING FLOWERING* genes and has strong bootstrap support (bs = 0.95) (Figure 5). Within this *FLC/MAF*-like group the safflower sequences grouped into a subclade with the sunflower *AGAMOUS*-like 31 MADS box gene. Hereafter we refer to Tr33367.4 and Tr33519.70 as safflower homologs of *MAF*-like genes, namely *CtMAF1* and *CtMAF2*, respectively.

We also used the *AtFLC* transcript as a search term using BLAST analysis within the safflower transcriptome and the closest hit was Tr32019, with two isoforms Tr32019.1 and Tr32019.2, but notably *AtFLC* did not hit upon any of the safflower candidate genes identified from the differential expression analysis. These Tr32019 isoforms were included in a new phylogenic analysis with members of the MAF- and FUL-clades (Supplementary Figure 6). Tr32019 groups with high confidence with sunflower *MAF1* and *MAF4*, which are themselves most closely related to *FLC* in Arabidopsis. Chicory, also a member of the Asteraceae with a vernalization response (Figure 1), has a functional homolog of *FLC*, namely *CiFL1* (Perilleux et al., 2013). We used *CiFL1* as a search term in a BLAST analysis of safflower transcripts and the closest hit was Tr32019, and not one of our candidate genes identified via differential gene expression analysis. We further examined the transcript abundance of Tr32019 in our RNASeq data and found there was no difference in expression in this transcript across a timecourse of vernalization nor between the two varieties S317 or C311 (Supplementary Figure 7). These analyses support the naming of Tr33367.4 and Tr33519.70 as *MAF* related genes and further work would be needed to see if either gene is a functional homolog of *AtFLC*.

### An Improved Reference Genome for Safflower

As part of an ongoing effort to improve the genomic resources in safflower we constructed a genome assembly for S317 using either Illumina short read technologies (∼100 bp reads) or PacBio long-read technologies (∼20,000 bp reads). Although both approaches assembled similar overall sequence lengths ∼1.1 Gb representing ∼80% of the predicted genome, summary data from PacBio-based compared to Illumina-based assembly showed significant improvements, including longer average length contigs (∼300,000 bp vs. 1,200 bp), reduced number of contigs (∼3,000 vs. 900,000) and a greatly improved N50 (∼600,000 bp vs. 2,000 bp) (Table 2). A generally accepted measure of genome completeness is provided by Benchmarking Universal Single-Copy Orthologs (BUSCO), where a core set of 1440 conserved genes across all known genomes is used to interrogate the completeness of an assembled genome (Simao et al., 2015). BUSCO metrics on the PacBio-based assembly indicate that the genome is at least 83% complete, with only 8% of the BUSCO core gene set missing (Table 3). The Illumina-based assembly was found to be at least 60% complete but has 22% of the BUSCO core gene set completely missing from the assembly. Overall, we found the PacBio-based assembly to be more complete, and less fragmented, relative to the Illumina-based analysis.

**TABLE 2:** Summary statistics for the *de novo* assemblies of safflower S317 using two different sequencing and software combinations.

Platform/assembler	Illumina/BioKanga	PacBio/Canu
Total size assembled (bp)	1,163,499,791	1,085,248,405
Contigs	904,199	3,565
Minimum contig length (bp)	300	14,010
N50 (bp)	1,940	594,302
Mean contig length (bp)	1,286	304,417
Maximum contig length (bp)	32,974	1,828,491

*See text for more details.*

**TABLE 3:** Assessment of Illumina- and PacBio-based assemblies using the BUSCO core gene set.

(a) *De novo* genome assembly using Illumina/BioKanga.		

BUSCOs searched	1440	%
Complete single-copy	863	60.0
Complete duplicated	39	2.7
Fragmented	222	15.4
Missing	316	21.9

**(b) *De novo* genome assembly using PacBio/Canu.**		

**BUSCOs searched**	**1440**	**%**

Complete single-copy	1206	83.8
Complete duplicated	88	6.1
Fragmented	35	2.4
Missing	111	7.7

*The assemblies were measured for completeness using the 1440 genes curated in the BUSCO (v3.0.1) protocol.*

### Construction of Gene Models for *CtFT1*, *CtFUL1*, *CtMAF1*, and *CtMAF2* Using a New Safflower Genome Assembly

**FIGURE 6:**
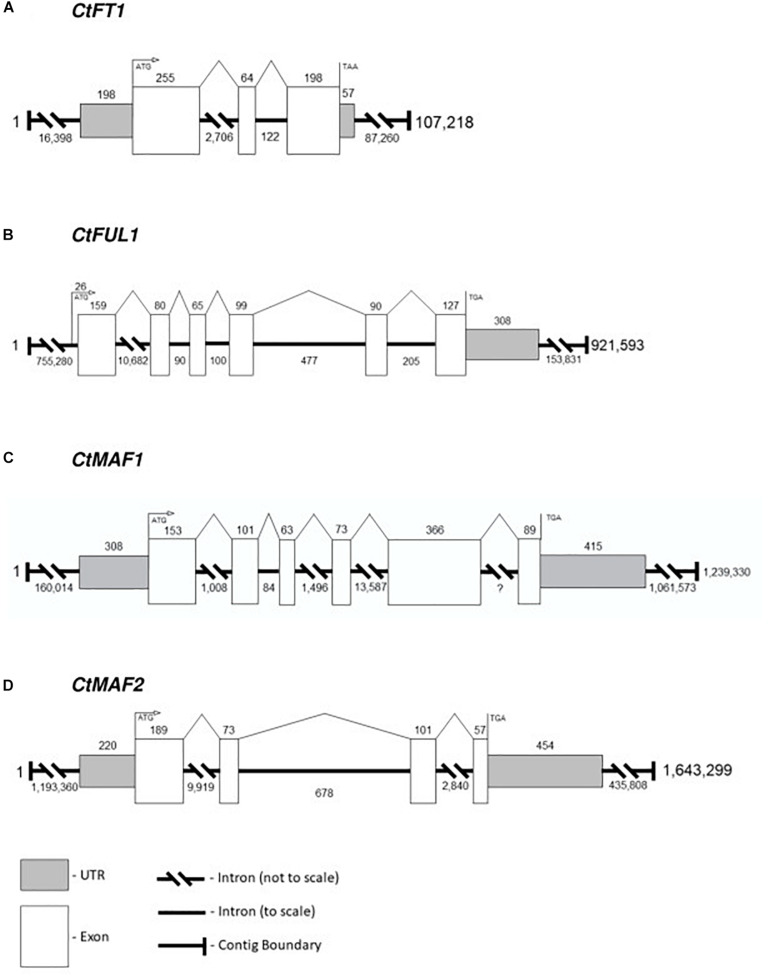
Gene models for **(A)** *CtFT1*, **(B)** *CtFUL1*, **(C)** *CtMAF1*, and **(D)** *CtMAF2* based on alignments of transcript with the genome assembly of S317.

Although there are many uses for a high-quality reference genome in safflower, in this report we limit ourselves to generating accurate gene models for four candidate genes identified from transcriptomic analysis, namely *CtFT1, CtFUL1, CtMAF1 and CtMAF2*. Each of the genes were confidently identified in the PacBio-based assembly. The structures of the four genes were assigned by aligning the transcripts of the genes against the DNA assembly, and therefore, the gene models are an accurate representation of exon-intron structures rather than predictions (Figure 6). *CtFT1* is found within a 107,218 bp contig and the gene contains two introns, the longest being 2,706 bp. *CtFUL1* is found within a contig that is 921,593 bp long and contains five introns, with the longest, intron 1, being over 10,000 bp in the first intron. *CtMAF1* is found on a contig being 1.2 Mbp long, contains five introns, including a 13,000 bp intron and an intron of undefined length, presumably due to the alignment needing to span two independent contigs. *CtMAF2* sits within a 1.6 Mb contig and contains three introns, the longest being 9,919 bp long.

### Genetics of Growth Habit in Safflower

Reciprocal crosses were conducted between C311 and S317 to test the genetic basis of growth habit in safflower. In total, 58 F1 plants were produced (20 F1 from C311♂xS317♀ and 38 F1 seed from S317♂xC311♀), and all displayed a rapid flowering ‘spring’ growth habit, indicating that spring growth habit is dominant. One F1 plant was self-pollinated generating a family of 142 F2 plants. Within this F2 family, 10 plants displayed a late flowering phenotype while the remaining 132 plants were early flowering. The observed frequency of late versus early flowering plants is consistent with a two gene model, with two unlinked dominant genes for spring-growth habit (expect 9 late and 133 early, χ^2^ = 0.73).

## Discussion

Here, we have shown that exposing imbibed safflower seeds to cold accelerates flowering when plants are subsequently grown in glasshouse conditions. This satisfies the formal definition of vernalization, that plants retain a memory of prolonged exposure to cold that accelerates flowering (Chouard, 1960). Similar to other plants, the vernalization response of safflower is quantitative, with longer exposure to cold accelerating flowering to greater extents, and seeds harvested from vernalized C311 exhibiting the same vernalization requirement as the parent, showing that the memory of cold is reset between generations. Similar to other species like *Arabidopsis* (Sheldon et al., 2008) and barley (Trevaskis et al., 2007) the need for vernalization is reset in a new generation. The requirement for vernalization was saturated after 2-weeks at 4 degrees, with no additional acceleration of flowering occurring with longer cold treatments. This response is rapid relative to some plants. For example, seeds of some cereals require 9–11 weeks of vernalization to saturate the vernalization response (Sasani et al., 2009). Based on the data presented here, we conclude that winter accessions of safflower possess a facultative vernalization response, such that vernalization accelerates flowering but plants are able to flower without vernalization. It should be noted that only one winter safflower accession has been studied here and that there are likely numerous other accessions possessing a vernalization requirement (Ghanavati and Knowles, 1977). Thus, there is potential that some winter safflower accessions might need longer periods of cold to fulfill the vernalization requirement.

Having established that winter-hardy safflower C311 has a vernalization response, we utilized an unbiased transcriptomics approach to investigate the molecular basis of this epigenetic pathway. This approach was based on those used previously to explore the molecular basis of vernalization in other plants. For example, microarray comparisons of gene expression in vernalized versus non-vernalized barley plants identified key genes that mediate the memory of vernalization amongst a relatively limited number of differentially expressed genes (Greenup et al., 2011). Similarly, gene-expression comparisons have proven to be an effective way to identify genes associated with the differences between spring versus winter cultivars of barley (Cuesta-Marcos et al., 2015). A key component of the experimental design was the sampling of tissue for vernalization treatments after allowing an extra week of growth at warm conditions. This approach takes advantage of the observation that genes involved in vernalization, as an epigenetic response, show lasting responses to prolonged cold and may contribute to the memory of winter (see Greenup et al., 2011). Additionally, genes that show lasting responses to vernalization and that are also differentially expressed between plants with winter and spring growth habit are of particular interest, since such genes potentially mediate both the vernalization response and reflect differences in vernalization requirement; as shown for *FLC* in Arabidopsis and *VRN1* in cereals. Finally, based on the dose-dependent response of vernalization we also visually inspected transcripts that also responded in a dose-dependent manner in C311. This experimental design and multi-layered analysis identified four transcripts, Tr32761.1, Tr26769.1, Tr33367.4 and Tr33519.70, that represent potential genes-of-interest with respect to the vernalization response of safflower. Through phylogenetic analyses we verified that Tr32761.1, Tr26769.1, Tr33367.4 and Tr33519.70 represent homologs of a *FT* gene, a *CUL*-like MADS-box gene and two closely related *FLC/MAF* MADS box genes. All of these safflower genes are quantitatively induced by vernalization, such that the increase in transcript levels in vernalized plants is proportional to the duration of the vernalization pre-treatment. Additionally, all these genes show elevated expression without vernalization in a spring versus winter safflower. Taken together, these findings strongly suggest that the expression of these genes is associated with the memory of vernalization in C311 and also the reduced vernalization requirement associated with spring growth habit in S317.

**FIGURE 7:**
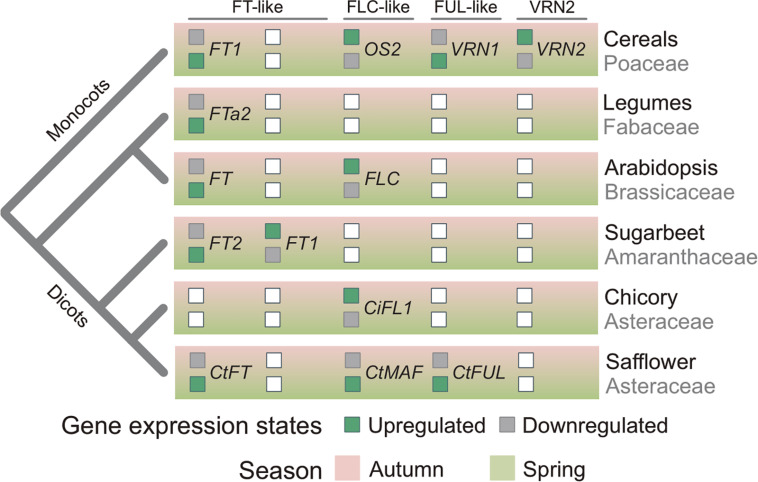
Relationships between phylogeny and seasonal gene expression signatures. Inferred seasonal gene-expression states of flowering regulators in plants related to phylogenetic relationships. Boxes filled green indicates whether a gene is actively expressed in either autumn or spring. Gray filled boxes indicate a gene is repressed in autumn or spring. White box denotes that the gene has not been found in a plant lineage or that the expression state is unknown. *CtMAF* refers to both *CtMAF1* and *CtMAF2*, which show similar seasonal gene expression patterns.

The data presented here, taken together with the findings of previous studies of vernalization-responsive genes, provides insights into the evolution of vernalization-induced flowering. Increased expression of *FT*-like genes in vernalized plants, relative to non-vernalized controls, has now been observed in diverse species, including monocots and all the major dicot lineages (Figure 7) (Michaels et al., 2005; Helliwell et al., 2006; Yan et al., 2006; Hemming et al., 2008; Laurie et al., 2011). This common pattern of transcriptional regulation observed across these diverse plant lineages suggests that limiting daylength activation of *FT* in non-vernalized plants is a common mechanism that delays flowering before winter, giving rise to the winter growth habit (vernalization requirement). This mechanism has potentially evolved independently in at least some lineages. For example, in sugar beet it appears that mutations have occurred recently in genes that regulate daylength flowering-responses and that these are a pre-requisite for the vernalization-responsive biennial/winter growth habit (Dally et al., 2014).

Elevated expression of a *FUL*-like gene (*VRN1*) in vegetative tissues following prolonged cold treatment is a distinctive feature of the vernalization response of cereals and related temperate grasses (McKeown et al., 2016). The elevated expression of *CtFUL1* (Tr26769.1) in vernalized safflower plants is similar to that observed for *VRN1* in cereals. Furthermore, *CtFUL1* showed elevated expression without vernalization in a spring safflower, relative to a winter accession. This pattern is also similar to the gene expression observed for the *VRN1* gene in cereals. This suggests that *FUL*-like genes might play a role in vernalization-induced flowering of both monocots and dicots. It is important to note here however, that there is a positive feedback loop between *FT*-like and *FUL*-like genes, such that increasing transcriptional activity of one class of gene can upregulate the other (Teper-Bamnolker and Samach, 2005; Deng et al., 2015). Since safflower *FUL1* and *FT1* genes have such similar gene expression patterns in response to vernalization, there is potential that either of these genes plays an active role in vernalization-induced flowering and that the other is up-regulated as a secondary response. This occurs in legumes, where *FUL*-like genes also show elevated expression in the vegetative tissues of vernalized plants, but are up-regulated by expression an *FT*-like gene as part of the long-day flowering response, rather than be induced directly by low temperatures as is the case for the *VRN1* gene of cereals (Jaudal et al., 2015). Assaying gene expression during seed vernalization, in darkness, could resolve whether safflower *FUL*-like or *FT*-like genes are directly regulated by cold.

Repression of *FLC* by vernalization is the central feature of vernalization-induced flowering of Arabidopsis and related *Brassicaceae* (Sheldon et al., 2000; Tadege et al., 2001; Irwin et al., 2016; O’Neill et al., 2019; Tudor et al., 2020; Yin et al., 2020). Genes related to *FLC* are also down regulated by vernalization in cereals and related grasses, likely downstream of the *VRN1* gene (Greenup et al., 2011; Ruelens et al., 2013; Deng et al., 2015). This study identified two safflower *MAF/FLC*-like genes, *CtMAF1* and *CtMAF2*, that are induced by vernalization. Induction of both genes was quantitative, with stronger induction occurring after longer vernalization pre-treatments, and both genes were expressed at elevated levels in spring versus winter safflower accessions. This is the inverse of the gene expression pattern observed for *FLC* in response to vernalization in Arabidopsis. Furthermore, it is unlike the pattern of *FLC* expression observed for winter versus spring ecotypes of Arabidopsis, with reduced expression of *MAF*-like genes in a winter safflower accession. The *MAF/FLC*-like group of MADS box genes appears to have expanded independently in *Asteraceae* and *Brassicaceae* (Figure 5). And so while the identified safflower genes are related to *FLC*, they are the most closely related orthologs and it is not possible to assume that these genes are direct functional equivalents. However, in the closely related Asteraceae species *Cichorium intybus*, a *FLC*-like gene, *CiFL1*, is the closest ortholog to the *A. thaliana FLC/MAF-*clade (Ruelens et al., 2013). *CiFL1* is induced by cold and represses flowering (Perilleux et al., 2013), suggesting functional conservation between *C. intybus* and *A. thaliana*. Interestingly, whereas *FLC* is repressed during longer cold treatments (>20 days) (Kim and Sung, 2013), *AtMAF4* and *AtMAF5* are induced by intermediate durations of cold (10–20 days), a result similar to the time course of *CtMAF1* and *CtMAF2* genes during the vernalization conditions examined here for safflower. Thus, it is possible that transcriptional activation by cold is a conserved feature of some *MAF/FLC*-like genes. Induction of *AtMAF4* and *AtMAF5* seems to mediate repression of flowering after short durations of vernalization, whereas induction of safflower *CtMAF/FLC*-like genes is associated with earlier flowering. As both safflower *MAF-like* genes identified here are also transcriptionally activated by low-temperature treatment, so this too appears to be a shared feature that is possibly conserved amongst some members of the *MAF/FLC-like* clade. Alternatively, low-temperature regulation of *MAF/FLC*-like genes might have evolved rapidly. Irrespective of evolutionary history, gene expression behavior of the Arabidopsis *FLC* gene cannot be assumed to be indicative of the broader *MAF/FLC*-like gene family.

Based on the data presented here, together with knowledge from other vernalization responsive plants, we suggest that vernalization-induced flowering has evolved on more than one occasion through the recruitment of common genes that are predisposed to function in seasonal flowering. These include the *FT*-like gene family, which are recruited to vernalization pathways when a requirement for cold evolves to override the daylength flowering response. Similarly, the conserved feedback loop between *FUL*-like and *FT*-like genes could lead to recruitment of *FUL*-like genes into vernalization pathways, either upstream (cereals) or downstream of *FT*-like genes (legumes). The *MAF/FLC-*like genes are potentially predisposed to recruitment to function in vernalization-response pathways through being temperature responsive, with evidence that this class of genes can be regulated by both cold and warm temperatures. There is also the possibility that conserved interactions between these genes led to their co-recruitment into vernalization dependent flowering; e.g., regulation of *FT*-like genes by *MAF/FLC*-like. A better understanding of global transcriptional responses of diverse plants to seasonal temperature and daylength cues, together with further functional analyses of these important classes of genes across diverse species could test these hypotheses in the future.

Variation in vernalization requirement, also described as growth habit, is an important trait in crop breeding since it adapts varieties to local seasonal conditions and management practices such as different sowing dates. Identification of genes controlling growth habit allows sequence-based approaches to be used to explore and capture genetic diversity for this trait. Molecular markers for genes controlling growth habit can then be used to facilitate parent selection in breeding crosses and also allow rapid selection of progeny. A key question arising from this study is: do any of the genes identified control safflower growth habit? The safflower spring growth habit is dominant to the winter growth-habit, such that F1 plants flower at the same time as the spring parent, and our genetic analysis suggested that S317 carries two independent genes for spring growth habit. One mechanism that could give rise to dominant genes for spring growth habit is gain-of-function in genes that activate flowering. Increased transcription is one mechanism that drives gain-of-function, so *CtFT1*, *CtFUL1*, *CtMAF1* and *CtMAF2* are possible candidates for the two genes controlling growth habit of safflower. Equally, mutations in a regulator of any of these genes, could give rise to a dominant spring growth habit.

This study generated a greatly improved genome sequence for safflower (S317 accession), and we used our assembled genome in combination with the transcriptome data to construct gene models for the candidate genes. These models will form the basis for future screening of diversity to determine the causal variation underlying the differences in responses to vernalization. Of particular interest are the first introns of *CtFUL1* and the *CtMAF1-2* genes of the MIKC-MADS box gene family. The first introns of MIKC-MADS box genes have been found to be key regions for the function genes controlling response to vernalization in other species. In Arabidopsis the first intron of *FLC* has been found to be essential for a stable repression by cold (Sheldon et al., 2002), and in barley and wheat, large deletions in the first intron of *VRN1*, also a MIKC-MADS box gene closely related to *AP1* (Yan et al., 2003), control the spring type growth thereby repressing the vernalization requirement (Fu et al., 2005).

Safflower has an estimated 1.35 Gb genome and the PacBio-based reference genome reported here is a significant improvement upon previously published resources, both in terms of completeness and length of contigs (Mayerhofer et al., 2011; Bowers et al., 2016). Nevertheless further improvements in the overall safflower genome can be made by incorporating all of the existing genome databases together generating a range of hybrid assemblies that together will improve the confidence in mapping long contigs to pseudo-chromosomes/linkage groupings and eventually building an even more complete genome.

In conclusion, this study demonstrates that a facultative requirement for vernalization is a feature of the winter growth habit of safflower. Transcriptome analyses identified a small list of safflower genes that are associated with both the memory of winter in vernalized plants and with differences in growth habit. Based on gene expression and sequence relationships with genes known to control vernalization-induced flowering and growth habit in other plants, these genes are interesting candidates for further research to explore the molecular basis of vernalization-induced flowering in safflower, which can provide insights into the evolution of vernalization as well as inform future crop improvement.

## Data Availability Statement

All sequencing data has been deposited in, and downloadable from, the CSIRO Data Access Portal (DAP):

Supplementary Dataset 1. Excel Spreadsheet for the calculations of data presented in Figure 3 and Supplementary Figure 3: https://doi.org/10.25919/611f-t870

Safflower S317 Genome Illumina Raw Reads and Assembly: https://doi.org/10.25919/xf81-3m21

Safflower S317 Genome PacBio Raw Reads: https://doi.org/10.25919/pbtp-kt44

Safflower S317 Genome PacBio Final Assembly: https://doi.org/10.25919/3jen-ht92

Safflower S317 Transcriptome Raw Reads: https://doi.org/10.25919/15sc-cn48

Safflower C311 and S317 Transcriptome Raw Reads during a vernalization time course: https://doi.org/10.25919/pqxw-f838

Safflower C311 transcriptome assembly: https://doi.org/10.25919/5jsa-p138.

## Author Contributions

DC, SF, BT, AE, and CW conceived and designed the experiments. DC conducted the experiments. DC and AS analyzed genomic data. DC and AS prepared data repositories for public release. DC, SF, BT, and CW analyzed the data. BT, SF, DC, AE, and CW wrote the manuscript. AE, BT, and CW wrote the funding applications. All authors contributed to the article and approved the submitted version.

## Conflict of Interest

The authors declare that the research was conducted in the absence of any commercial or financial relationships that could be construed as a potential conflict of interest.

## Footnotes

1 https://github.com/csiro-crop-informatics/biokanga

2 www.genelink.com/tools/gl-oe.asp

3 www.premierbiosoft.com/netprimer/
