# Supplementary material for: A Vernalization Response in a Winter Safflower (Carthamus tinctorius) Involves the Upregulation of Homologs of FT, FUL, and MAF
Source: Front Plant Sci. 2021 Mar 30;12:639014. doi: 10.3389/fpls.2021.639014 (PMC8043130; doi:10.3389/fpls.2021.639014)
Supplement: Supplementary file 1 [file Image_1.pdf]

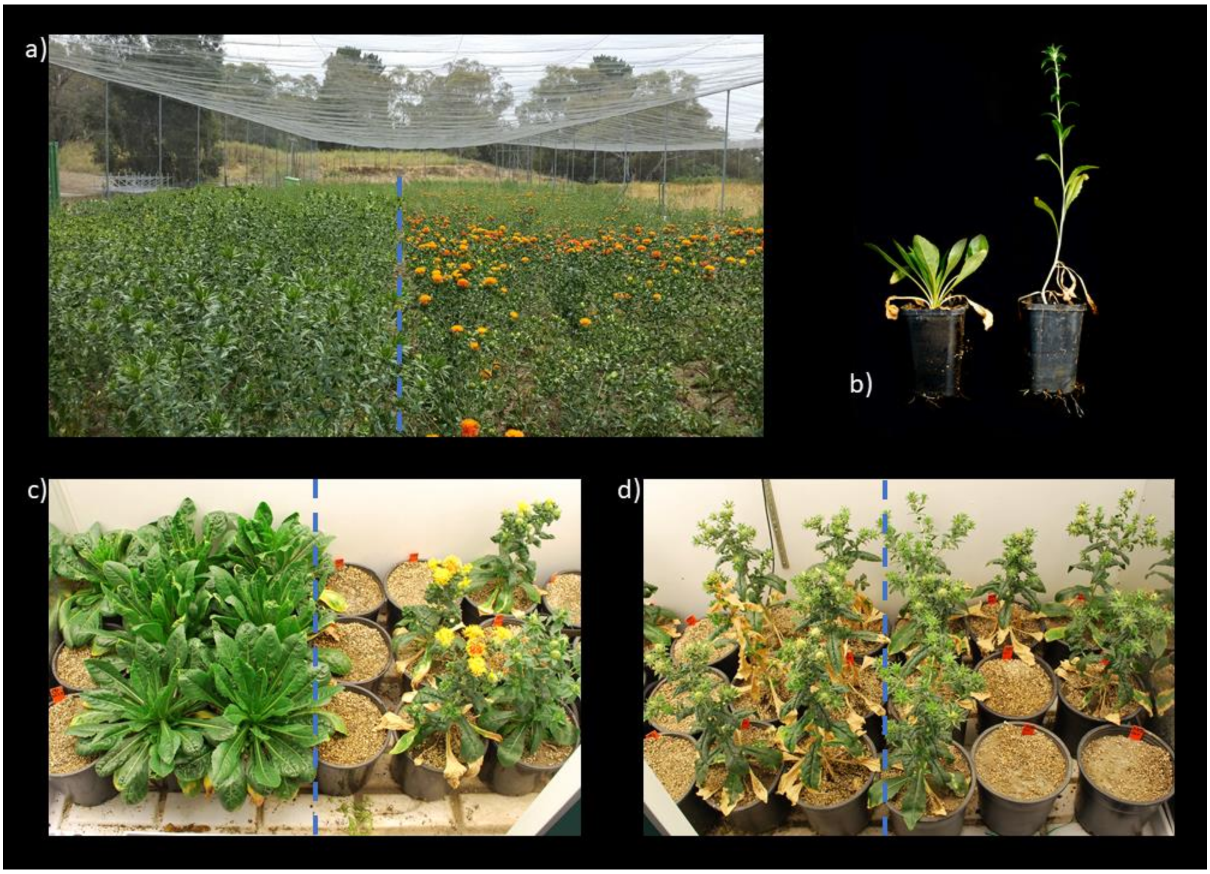

SuppFigure1: Comparisons of the growth behaviour of spring (S317) and winter (C311) safflower. (a): A field trial at a small plot site at CSIRO Black Mountain, Canberra, Australia, growing C311 (left) and S317. The field was sown early spring. (b): C311 plants, 6 weeks old, unvernalized (left) and vernalized (right), grown in long day growth conditions. (c) C311 plants grown without any cold treatment (left hand side of the panel) or with a 15 day cold treatment (right hand side of the panel); (d) S317 plants, 7 weeks old, grown either without any cold treatment (left side of the panel) and with a 15 day cold treatment (right side of the panels). In panels b, c and d the plants were exposed to long day conditions.
