# Supplementary material for: A Vernalization Response in a Winter Safflower (Carthamus tinctorius) Involves the Upregulation of Homologs of FT, FUL, and MAF
Source: Front Plant Sci. 2021 Mar 30;12:639014. doi: 10.3389/fpls.2021.639014 (PMC8043130; doi:10.3389/fpls.2021.639014)
Supplement: Supplementary file 2 [file Image_2.pdf]

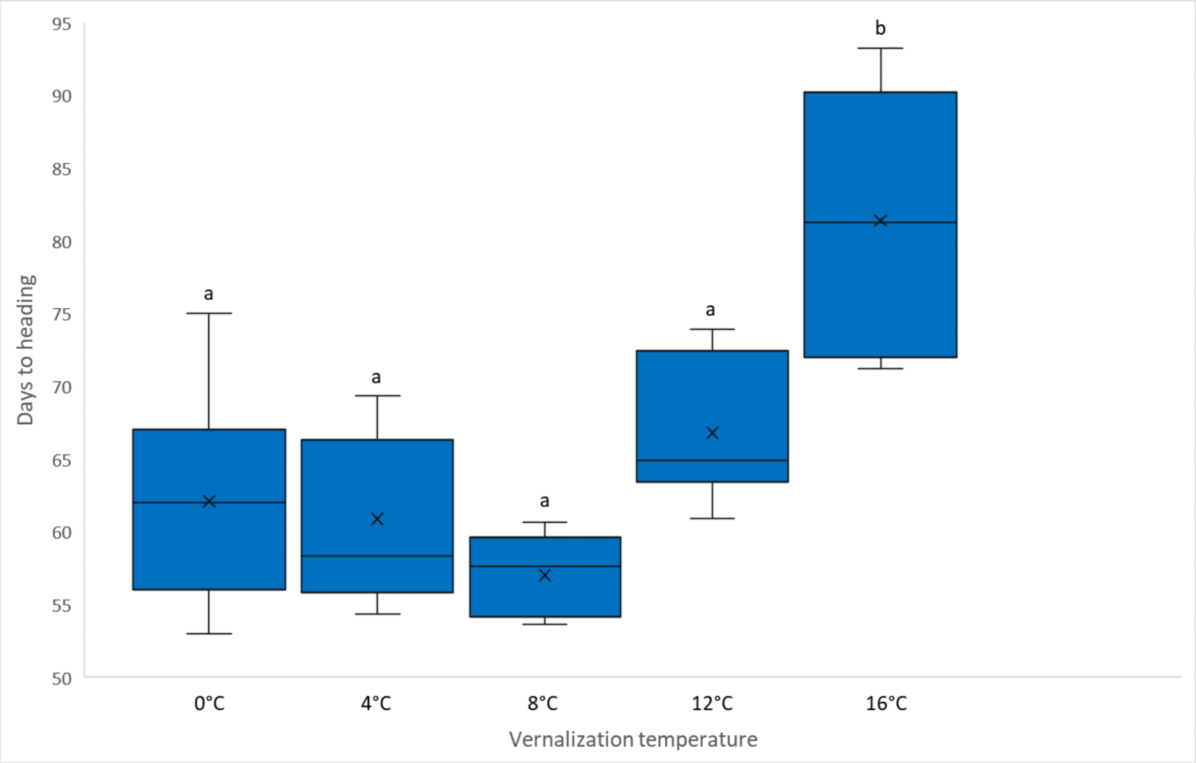

Supplementary Figure 2: Effect of vernalization for 20 days at different temperatures on the heading date for C311. Data summaries are upper and lower quartile (blue boxes) and the range covering 95% of the data (further extensions), “X” indicates the mean for each set with 10 plants per treatment. Analysis with an ANOVA and Tukey HSD post-hoc analysis showed there were no significant differences in heading date at vernalization temperatures below 12°C (as marked with ‘a’), however, vernalization at 16°C (as marked with ‘b’) showed a significantly longer time to heading when compared to the lower temperatures.
