# Supplementary material for: A Vernalization Response in a Winter Safflower (Carthamus tinctorius) Involves the Upregulation of Homologs of FT, FUL, and MAF
Source: Front Plant Sci. 2021 Mar 30;12:639014. doi: 10.3389/fpls.2021.639014 (PMC8043130; doi:10.3389/fpls.2021.639014)
Supplement: Supplementary file 3 [file Image_3.pdf]

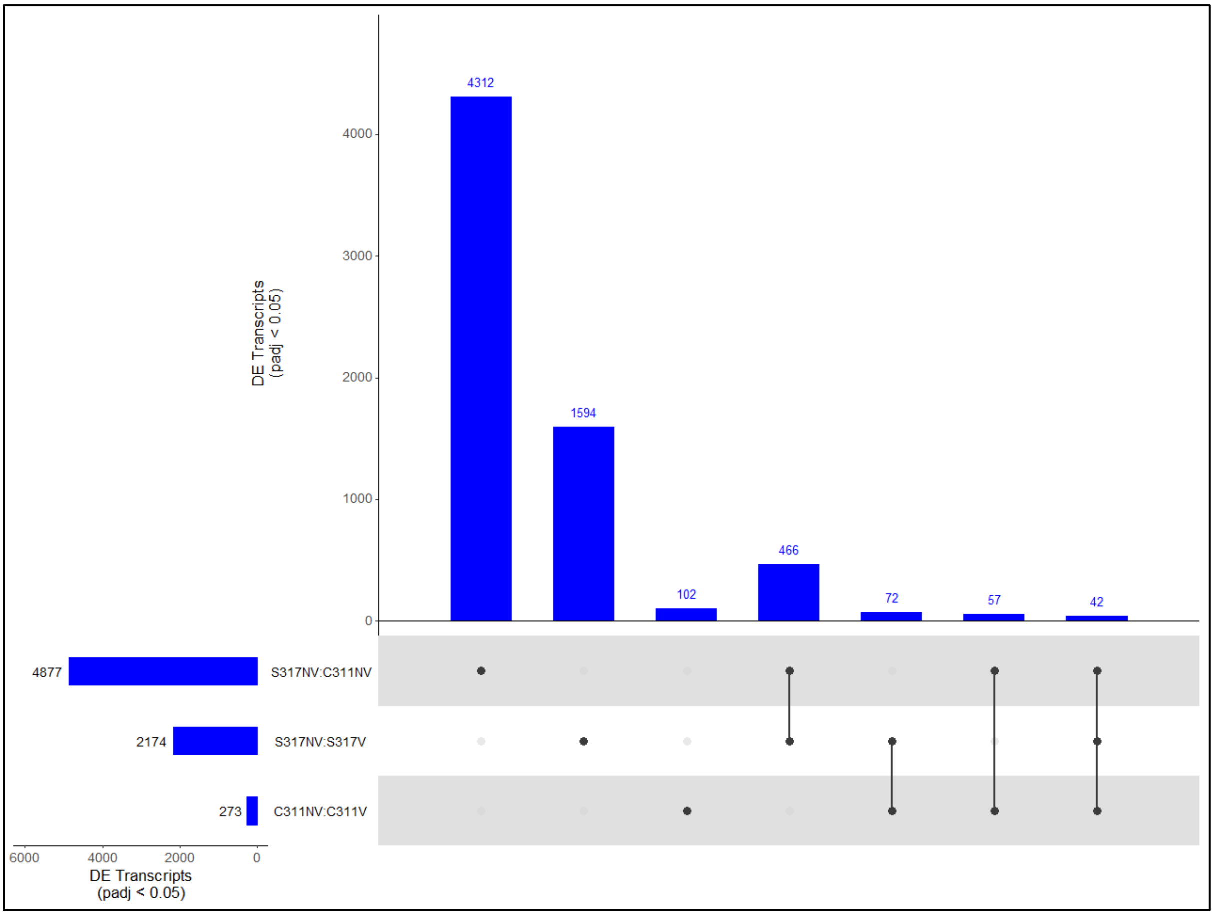

SuppFigure 3: UpSet plot showing the number of significantly differentially expressed transcripts (adjusted p-value < 0.05) in different groupings, including comparisons between unvernalized S317 against unvernalized C311 (S317NV:C311NV), S317 unvernalized vs vernalised (S317NV:S317V), and C311 unvernalized vs vernalized (C311NV:C311V). Rows show total number of significantly differentially expressed transcripts for each comparison, columns show significantly differentially expressed transcripts in each interaction.
