# Supplementary material for: A Vernalization Response in a Winter Safflower (Carthamus tinctorius) Involves the Upregulation of Homologs of FT, FUL, and MAF
Source: Front Plant Sci. 2021 Mar 30;12:639014. doi: 10.3389/fpls.2021.639014 (PMC8043130; doi:10.3389/fpls.2021.639014)
Supplement: Supplementary file 4 [file Image_4.pdf]

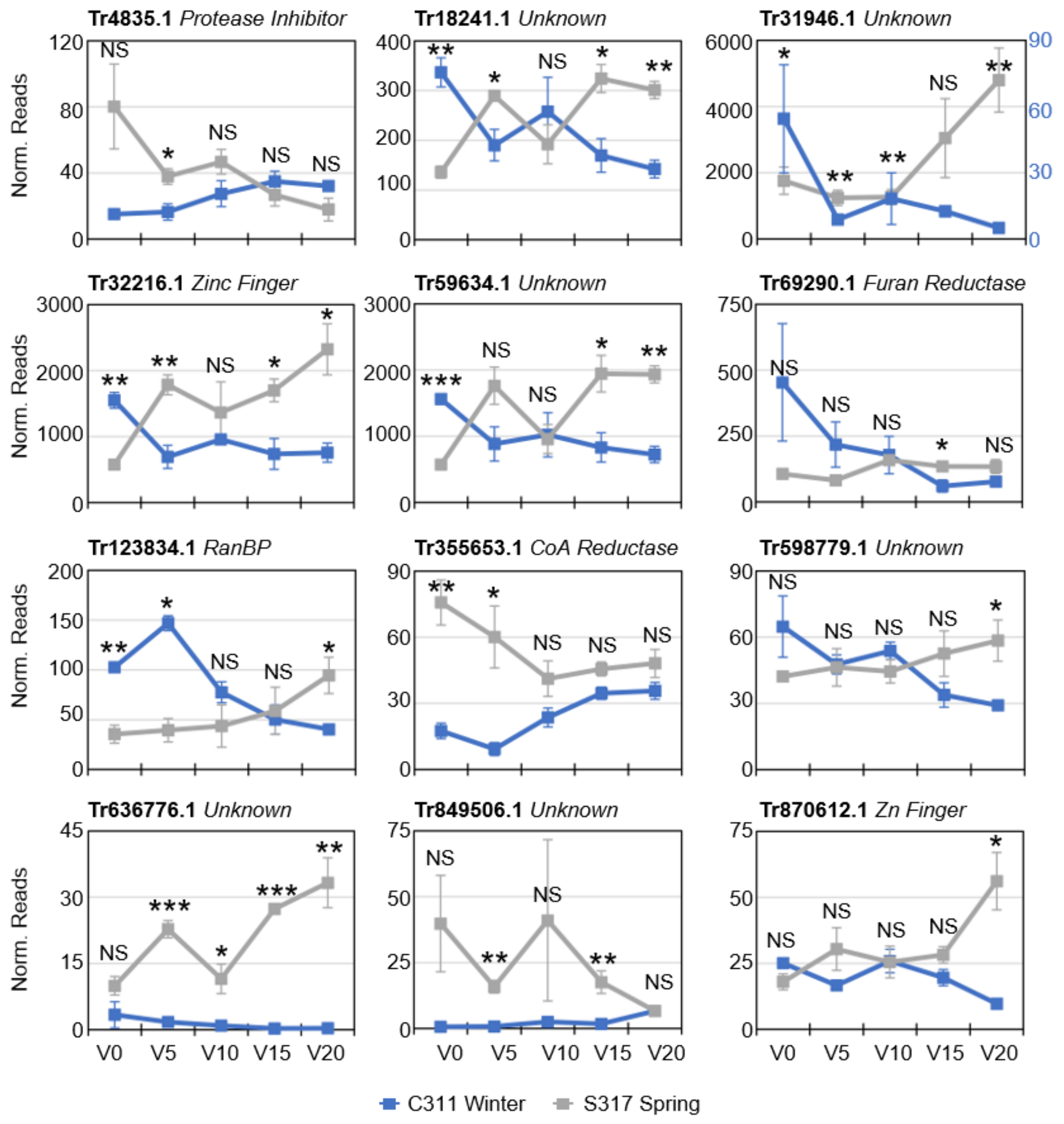

Supplementary Figure 4. Gene expression patterns of transcripts identified as differentially expressed during vernalization treatments but did not exhibit a dose dependent response to vernalization in the winter safflower C311. Plotted on the y-axes are the average normalized transcript read counts, from 3 biological replicates for key transcripts from transcriptomes of non-vernalized plants (V0) or plants that had been vernalized for 5, 10, 15 or 20 days. Data are presented for the winter safflower accession (C311, blue line), contrasted with the spring cultivar (S317, grey line). Error bars show standard error. Statistical tests include Student's t-test comparison to the non-vernalized control for time course analysis of expression in the winter safflower (when plotted alone) or comparison between the spring versus winter safflower when genotypes are compared (NS = non-significant, * $P<0.05$, ** $P<0.01$, *** $P<0.001$). Data are not presented for Tr72407.1, which is likely derived from the same gene as Tr69290.1 and showed a near identical pattern of gene expression. Similarly, Tr33367.1 seems to be derived from the same gene as Tr33367.4 (*CtMAF1*) and so is not presented.
