# Supplementary material for: A Vernalization Response in a Winter Safflower (Carthamus tinctorius) Involves the Upregulation of Homologs of FT, FUL, and MAF
Source: Front Plant Sci. 2021 Mar 30;12:639014. doi: 10.3389/fpls.2021.639014 (PMC8043130; doi:10.3389/fpls.2021.639014)
Supplement: Supplementary file 5 [file Image_5.pdf]

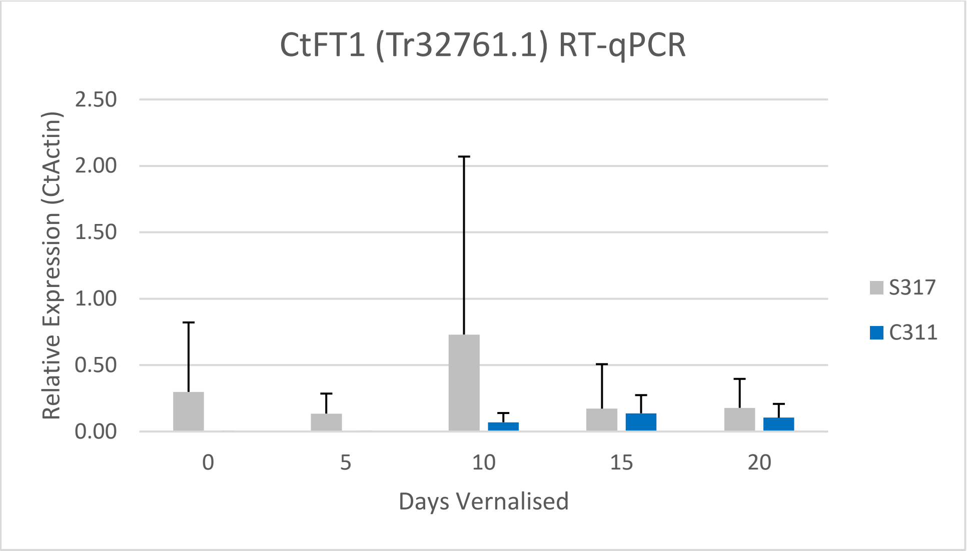

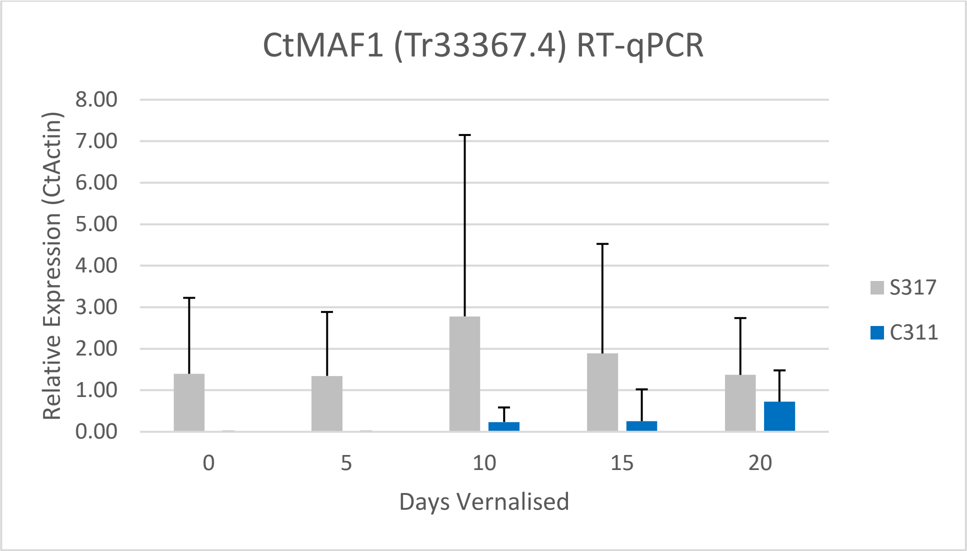

Supplementary Figure 5. RT-qPCR expression analysis of transcripts for *CtFT1* (upper panel) and *CtMAF1* (lower panel), relative to the expression of *CtActin*. The sequence of primers used are found in Supplementary Table 3, other technical and method details can be found in the main text.
