# Supplementary material for: A Vernalization Response in a Winter Safflower (Carthamus tinctorius) Involves the Upregulation of Homologs of FT, FUL, and MAF
Source: Front Plant Sci. 2021 Mar 30;12:639014. doi: 10.3389/fpls.2021.639014 (PMC8043130; doi:10.3389/fpls.2021.639014)
Supplement: Supplementary file 6 [file Image_6.pdf]

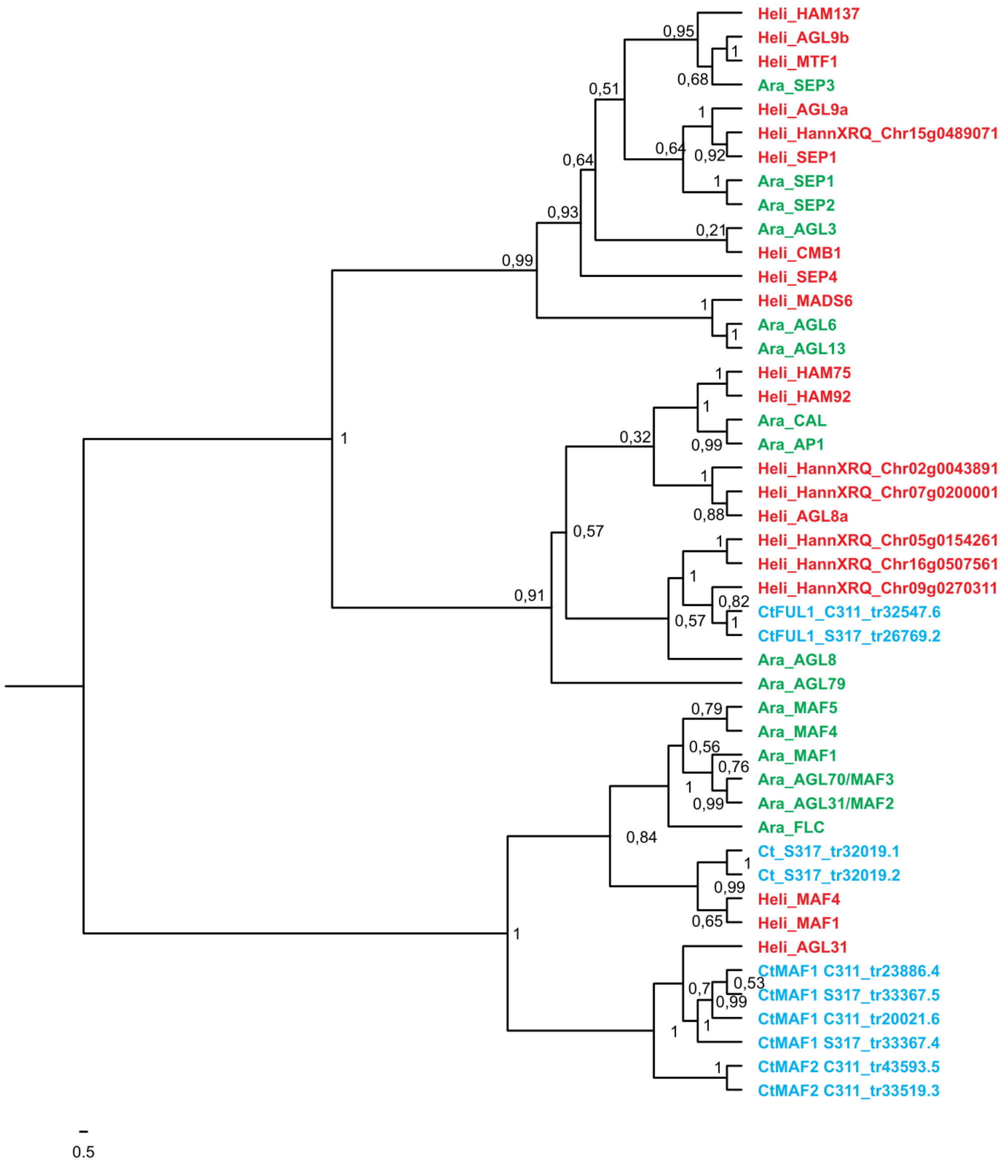

Supplementary Figure 6. Maximum likelihood phylogeny of the *MAF*- and *FUL*- clades of the MIKC MADS gene family in *Arabidopsis thaliana* (in green) and *Helianthus annum* (in red). Transcripts of *Carthamus tinctorius* (in blue) found to be differentially expressed in response to vernalization were included, as well as two transcripts Ct_S317_tr32019.1 and .2 that were identified as the best hits in a BLAST search of *A. thaliana FLC* to the transcriptome of S317 and also in a BLAST using chicory *CiFL1*. Node values are bootstrap support values. Information about the genes can be found in Supplementary table 1.
