# Supplementary material for: A Vernalization Response in a Winter Safflower (Carthamus tinctorius) Involves the Upregulation of Homologs of FT, FUL, and MAF
Source: Front Plant Sci. 2021 Mar 30;12:639014. doi: 10.3389/fpls.2021.639014 (PMC8043130; doi:10.3389/fpls.2021.639014)
Supplement: Supplementary file 7 [file Image_7.pdf]

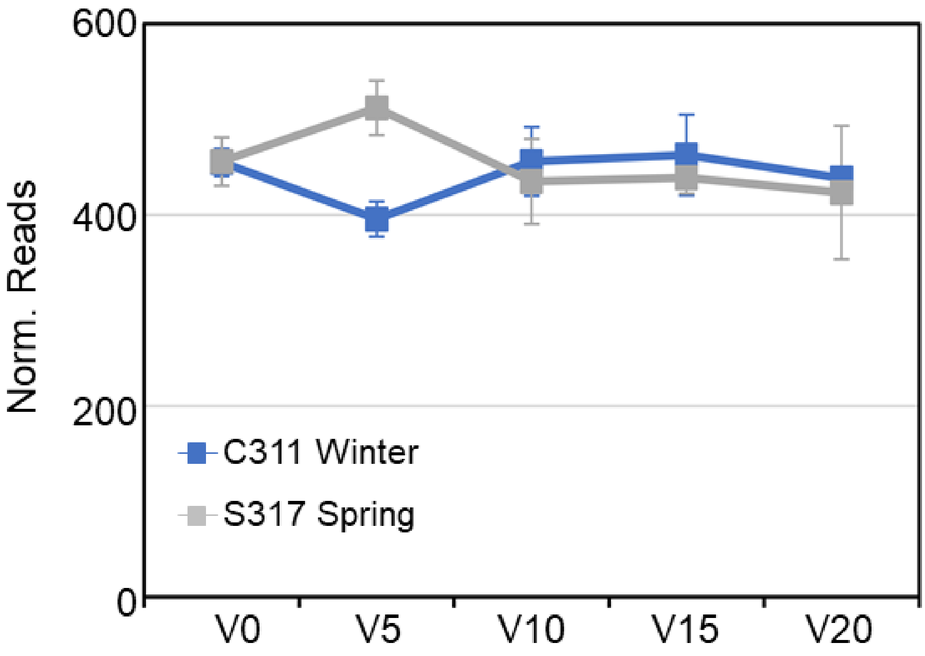

SuppFigure7. Level of transcript abundance for Tr32019 in safflower S317 and C311 during a time course of vernalization

Tr32019.1 is a safflower gene closely related to chicory *CiFL1*. Plotted on the y-axes are the average normalized transcript read counts, from 3 biological replicates for Tr32019.1 (CarTin_tx_s317_comp32019_c1_seq1) from transcriptomes of non-vernalized plants (V0) versus plants that had been vernalized for 5, 10, 15 or 20 days. Data are presented for the winter safflower accession (C311, blue line) versus the spring cultivar (S317, grey line). There was no difference in transcript levels between vernalized and non-vernalized control plants for either accession, at any timepoint (T-TEST, $p>0.05$ all comparisons).
