# Supplementary material for: A Vernalization Response in a Winter Safflower (Carthamus tinctorius) Involves the Upregulation of Homologs of FT, FUL, and MAF
Source: Front Plant Sci. 2021 Mar 30;12:639014. doi: 10.3389/fpls.2021.639014 (PMC8043130; doi:10.3389/fpls.2021.639014)
Supplement: Supplementary file 8 [file Table_1.pdf]

**Supplementary Table S1**. Primers used for RT-qPCR

| Transcript | Direction | Sequence |
|---|---|---|
| *CtActin* | Forward | 5’-ACCACAGGTATTGTGCTGGATTC-3’ |
| | Reverse | 5’-CACCAATTGTGATGACTTGTCCAT-3’ |
| *CtFT1* (Tr32761.1) | Forward | 5’-AGCGACCACAGGAACACG-3’ |
| | Reverse | 5’-GGAACAAACACGAAAACCATACG-3’ |
| *CtMAF1* (Tr33367.4) | Forward | 5’-CTCCAACAAGTCAGAACCAGAAAG-3’ |
| | Reverse | 5’-CAGCCTCAACCATCATCAGC-3’ |
