# Supplementary material for: A Vernalization Response in a Winter Safflower (Carthamus tinctorius) Involves the Upregulation of Homologs of FT, FUL, and MAF
Source: Front Plant Sci. 2021 Mar 30;12:639014. doi: 10.3389/fpls.2021.639014 (PMC8043130; doi:10.3389/fpls.2021.639014)
Supplement: Supplementary file 9 [file Table_2.pdf]

Supplementary Table S2. References to sequences used for phylogenetic analysis of the FT and MADS box gene families from *Arabidopsis thaliana* and *Helianthus annuus*.

Species	Gene family	Genome locus	Gene	Protein	pfam identifier
Arabidopsis thaliana	MADS-box	At1g01530	AGL28	Agamous-like MADS-box protein AGL28	Q9LMM8
Arabidopsis thaliana	MADS-box	At1g17310	At1g17310	MADS-box transcription factor family protein	A0A1P8AT79
Arabidopsis thaliana	MADS-box	At1g18750	AGL65	AGAMOUS-like 65	A0A1P8AR61
Arabidopsis thaliana	MADS-box	At1g22130	AGL104	Agamous-like MADS-box protein AGL104	Q9LM46
Arabidopsis thaliana	MADS-box	At1g22590	AGL87	AGAMOUS-like 87	Q7X9H1
Arabidopsis thaliana	MIKC MADS-box	At1g24260	SEP3	Developmental protein SEPALLATA 3	O22456
Arabidopsis thaliana	MIKC MADS-box	At1g26310	CAL	Transcription factor CAULIFLOWER	Q39081
Arabidopsis thaliana	MADS-box	At1g28450	AGL58	AGAMOUS-like 58	Q9SGP3
Arabidopsis thaliana	MADS-box	At1g28460	AGL59	AGAMOUS-like 59	Q9SGP4
Arabidopsis thaliana	MADS-box	At1g29962	AGL64	AGAMOUS-like 64	Q7XJK9
Arabidopsis thaliana	MIKC MADS-box	At1g31140	AGL63	Agamous-like MADS-box protein AGL63	Q9SA07
Arabidopsis thaliana	MADS-box	At1g31630	AGL86	Agamous-like MADS-box protein AGL86	Q9C6V3
Arabidopsis thaliana	MADS-box	At1g31640	AGL92	Agamous-like MADS-box protein AGL92	Q9C6V4
Arabidopsis thaliana	MADS-box	At1g33070	At1g33070	MADS-box family protein	F4HPG8
Arabidopsis thaliana	MADS-box	At1g46408	AGL97	Agamous-like MADS-box protein AGL97	Q9C633
Arabidopsis thaliana	MADS-box	At1g47760	AGL102	AGAMOUS-like 102	Q9FZF2
Arabidopsis thaliana	MADS-box	At1g48150	At1g48150	F21D18.12	Q9LNG8
Arabidopsis thaliana	MADS-box	At1g54760	AGL85	AGAMOUS-like 85	Q9ZVL8
Arabidopsis thaliana	MADS-box	At1g59810	AGL50	AGAMOUS-like 50	Q9XIE7
Arabidopsis thaliana	MADS-box	At1g60040	AGL49	Agamous-like MADS-box protein AGL49	Q9ZUI9
Arabidopsis thaliana	MADS-box	At1g60880	AGL56	AGAMOUS-like-56	Q9C963
Arabidopsis thaliana	MADS-box	At1g60920	AGL55	AGAMOUS-like 55	Q9C960
Arabidopsis thaliana	MADS-box	At1g65300	PHE2	MADS-box transcription factor PHERES 2	Q7XJK8
Arabidopsis thaliana	MADS-box	At1g65330	PHE1	MADS-box transcription factor PHERES 1	O80805
Arabidopsis thaliana	MADS-box	At1g65360	AGL23	Agamous-like MADS-box protein AGL23	O80807
Arabidopsis thaliana	MIKC MADS-box	At1g69120	AP1	Floral homeotic protein APETALA 1	P35631
Arabidopsis thaliana	MADS-box	At1g69540	AGL94	AGAMOUS-like 94	A0A1P8ASZ9
Arabidopsis thaliana	MIKC MADS-box	At1g71692	AGL12	Agamous-like MADS-box protein AGL12	Q38841
Arabidopsis thaliana	MADS-box	At1g72350	At1g72350	At1g72350	Q9C9D4
Arabidopsis thaliana	MIKC MADS-box	At1g77080	MAF1	K-box region and MADS-box transcription factor family protein	A0A1P8ARA3
Arabidopsis thaliana	MADS-box	At1g77950	AGL67	AGAMOUS-like 67	F4I8L6

Arabidopsis thaliana	MADS-box	At1g77980	AGL66	Agamous-like MADS-box protein AGL66	Q1PFC2
Arabidopsis thaliana	MADS-box	At2g03060	AGL30	Agamous-like MADS-box protein AGL30	Q1PFA4
Arabidopsis thaliana	MIKC MADS-box	At2g03710	AGL3	Agamous-like MADS-box protein AGL3	P29383
Arabidopsis thaliana	MIKC MADS-box	At2g14210	agl44	AGAMOUS-like 44	A0A1I9LM26
Arabidopsis thaliana	MIKC MADS-box	At2g22540	SVP	MADS-box protein SVP	Q9FVC1
Arabidopsis thaliana	MIKC MADS-box	At2g22630	AGL17	Agamous-like MADS-box protein AGL17	Q38840
Arabidopsis thaliana	MADS-box	At2g24840	AGL61	Agamous-like MADS-box protein AGL61	Q4PSU4
Arabidopsis thaliana	MADS-box	At2g26320	AGL33	AGAMOUS-like 33	O64840
Arabidopsis thaliana	MADS-box	At2g28700	AGL46	AGAMOUS-like 46	F4IIT6
Arabidopsis thaliana	MADS-box	At2g34440	AGL29	Agamous-like MADS-box protein AGL29	O64703
Arabidopsis thaliana	MADS-box	At2g40210	AGL48	AGAMOUS-like 48	Q9XEF1
Arabidopsis thaliana	MIKC MADS-box	At2g42830	AGL5	Agamous-like MADS-box protein AGL5	P29385
Arabidopsis thaliana	MIKC MADS-box	At2g45650	AGL6	Agamous-like MADS-box protein AGL6	P29386
Arabidopsis thaliana	MIKC MADS-box	At2g45660	SOC1	MADS-box protein SOC1	O64645
Arabidopsis thaliana	MIKC MADS-box	At3g02310	SEP2	Developmental protein SEPALLATA 2	P29384
Arabidopsis thaliana	MADS-box	At3g04100	AGL57	AGAMOUS-like 57	Q9M8W6
Arabidopsis thaliana	MADS-box	At3g05860	At3g05860	MADS-box transcription factor family protein	F4J9K3
Arabidopsis thaliana	MADS-box	At3g18650	AGL103	Agamous-like MADS-box protein AGL103	Q9LSB2
Arabidopsis thaliana	MIKC MADS-box	At3g30260	AGL79	AGAMOUS-like 79	Q7X9H6
Arabidopsis thaliana	MIKC MADS-box	At3g54340	AP3	Floral homeotic protein APETALA 3	P35632
Arabidopsis thaliana	MIKC MADS-box	At3g57230	AGL16	Agamous-like MADS-box protein AGL16	A2RVQ5
Arabidopsis thaliana	MIKC MADS-box	At3g57390	AGL18	Agamous-like MADS-box protein AGL18	Q9M2K8
Arabidopsis thaliana	MIKC MADS-box	At3g58780	SHP1	K-box region and MADS-box transcription factor family protein	F4J705
Arabidopsis thaliana	MIKC MADS-box	At3g61120	AGL13	Agamous-like MADS-box protein AGL13	Q38837
Arabidopsis thaliana	MADS-box	At3g66656	AGAMOUS-like 91	AGAMOUS-like 91	Q9C836
Arabidopsis thaliana	MADS-box	At4g02235	T2H3.15	AGAMOUS-like 51	O81421
Arabidopsis thaliana	MIKC MADS-box	At4g09960	stk	K-box region and MADS-box transcription factor family protein	F4JKV2
Arabidopsis thaliana	MADS-box	At4g11250	AGL52	AGAMOUS-like 52	Q9SUT6
Arabidopsis thaliana	MIKC MADS-box	At4g11880	AGL14	Agamous-like MADS-box protein AGL14	Q38838

Arabidopsis thaliana	MIKC MADS-box	At4g18960	AG	Floral homeotic protein AGAMOUS	P17839
Arabidopsis thaliana	MIKC MADS-box	At4g22950	AGL19	Agamous-like MADS-box protein AGL19	O82743
Arabidopsis thaliana	MIKC MADS-box	At4g24540	AGL24	MADS-box protein AGL24	O82794
Arabidopsis thaliana	MADS-box	At4g36590	C7A10.770	MADS-box protein	O23222
Arabidopsis thaliana	MADS-box	At4g37435	At4g37435	MADS-box transcription factor family protein	A0A1P8 B5J4
Arabidopsis thaliana	MIKC MADS-box	At4g37940	AGL21	Agamous-like MADS-box protein AGL21	Q9SZJ6
Arabidopsis thaliana	MADS-box	At5g04640	T32M21_240	AGAMOUS-like 99	Q9LZ61
Arabidopsis thaliana	MADS-box	At5g06500	AGL96	AGAMOUS-like 96	Q9FG20
Arabidopsis thaliana	MIKC MADS-box	At5g10140	FLC	MADS-box protein FLOWERING LOCUS C	Q9S7Q7
Arabidopsis thaliana	MIKC MADS-box	At5g13790	AGL15	AGAMOUS-like 15	A0A1P8 BA33
Arabidopsis thaliana	MIKC MADS-box	At5g15800	SEP1	Developmental protein SEPALLATA 1	P29382
Arabidopsis thaliana	MIKC MADS-box	At5g20240	PI	Floral homeotic protein PISTILLATA	P48007
Arabidopsis thaliana	MIKC MADS-box	At5g23260	TT16	Protein TRANSPARENT TESTA 16	Q8RYD 9
Arabidopsis thaliana	MADS-box	At5g26580	AGL34	AGAMOUS-like-34	Q7X9H2
Arabidopsis thaliana	MADS-box	At5g26630	At5g26630	MADS-box protein AGL35	Q7XJK7
Arabidopsis thaliana	MADS-box	At5g26650	AGL36	Agamous-like MADS-box protein AGL36	Q7XJK6
Arabidopsis thaliana	MADS-box	At5g26865	F2P16.19	AGAMOUS-like MADS-box protein	O04632
Arabidopsis thaliana	MADS-box	At5g26950	AGL93	Agamous-like MADS-box protein AGL93	Q7X9H9
Arabidopsis thaliana	MADS-box	At5g27050	AGL101	AGAMOUS-like 101	Q4PSE3
Arabidopsis thaliana	MADS-box	At5g27070	AGL53	Agamous-like MADS-box protein AGL53	Q7X9N2
Arabidopsis thaliana	MADS-box	At5g27090	AGL54	AGAMOUS-like 54	Q9S9U2
Arabidopsis thaliana	MADS-box	At5g27130	AGL39	AGAMOUS-like 39	F4K2U3
Arabidopsis thaliana	MADS-box	At5g27580	AGL89	AGAMOUS-like 89	Q7XJL1
Arabidopsis thaliana	MADS-box	At5g27810	At5g27810	At5g27810	Q3E8Z9
Arabidopsis thaliana	MADS-box	At5g27944	At5g27944	MADS-box transcription factor family protein	B3H5S0
Arabidopsis thaliana	MADS-box	At5g27960	AGL90	Agamous-like MADS-box protein AGL90	Q7XJK5
Arabidopsis thaliana	MADS-box	At5g37415	AGL105	AGAMOUS-like 105	B3H4N9
Arabidopsis thaliana	MADS-box	At5g38620	MBB18.17	MADS-box protein AGL73	Q9FFV9
Arabidopsis thaliana	MADS-box	At5g38740	AGL77	AGAMOUS-like 77	Q9FKR2
Arabidopsis thaliana	MADS-box	At5g39750	AGL81	Agamous-like MADS-box protein AGL81	Q9FIX0
Arabidopsis thaliana	MADS-box	At5g39810	AGL98	AGAMOUS-like 98	Q9FIW6
Arabidopsis thaliana	MADS-box	At5g40120	AGL76	AGAMOUS-like 76	Q9FL19

Arabidopsis thaliana	MADS-box	At5g40220	AGL43	AGAMOUS-like 43	Q9FL10
Arabidopsis thaliana	MADS-box	At5g41200	AGL75	Agamous-like MADS-box protein AGL75	Q9FLL0
Arabidopsis thaliana	MADS-box	At5g48670	AGL80	Agamous-like MADS-box protein AGL80	Q9FJK3
Arabidopsis thaliana	MADS-box	At5g49420	At5g49420	MADS-box protein	Q7X9H5
Arabidopsis thaliana	MADS-box	At5g49490	AGL83	AGAMOUS-like 83	Q9FGZ5
Arabidopsis thaliana	MIKC MADS-box	At5g51860	AGL72	MADS-box protein AGL72	Q9FLH5
Arabidopsis thaliana	MIKC MADS-box	At5g51870	AGL71	AGAMOUS-like 71	F4KEP6
Arabidopsis thaliana	MADS-box	At5g55690	MDF20.13	At5g55690	Q9FM69
Arabidopsis thaliana	MADS-box	At5g58890	AGL82	Agamous-like MADS-box protein AGL82	Q9FIM0
Arabidopsis thaliana	MADS-box	At5g60440	AGL62	Agamous-like MADS-box protein AGL62	Q9FKK2
Arabidopsis thaliana	MIKC MADS-box	At5g60910	FUL	Agamous-like MADS-box protein AGL8	Q38876
Arabidopsis thaliana	MIKC MADS-box	At5g62165	AGL42	MADS-box protein AGL42	Q9FIS1
Arabidopsis thaliana	MIKC MADS-box	At5g65050	AGL31/MAF2	Agamous-like MADS-box protein AGL31	Q9FPN7
Arabidopsis thaliana	MIKC MADS-box	At5g65060	AGL70/MAF3	Agamous-like MADS-box protein AGL70	Q9LSR7
Arabidopsis thaliana	MIKC MADS-box	At5g65070	MAF4	K-box region and MADS-box transcription factor family protein	Q9LSR6
Arabidopsis thaliana	MIKC MADS-box	At5g65080	MAF5	K-box region and MADS-box transcription factor family protein	A0A2H1ZE96
Arabidopsis thaliana	MADS-box	At5g65330	AGL78	AGAMOUS-like 78	Q9FKQ5
Helianthus annuus	MIKC MADS-box	HannXRQ_Chr00c0029g0571181	HAM137	MADS-box transcriptional factor HAM137	Q84LB9
Helianthus annuus	MIKC MADS-box	HannXRQ_Chr01g0007431	HannXRQ_Chr01g0007431	Putative transcription factor, MADS-box, Transcription factor, K-box	A0A251VNY4
Helianthus annuus	MIKC MADS-box	HannXRQ_Chr01g0007901	HannXRQ_Chr01g0007901	Putative transcription factor, MADS-box	A0A251VN10
Helianthus annuus	MIKC MADS-box	HannXRQ_Chr01g0015681	AGL15	Putative agamous-like MADS-box protein AGL15	A0A251VNJ7
Helianthus annuus	MADS-box	HannXRQ_Chr01g0018311	HannXRQ_Chr01g0018311	Putative transcription factor, MADS-box	A0A251VRM4
Helianthus annuus	MADS-box	HannXRQ_Chr01g0018361	HannXRQ_Chr01g0018361	Putative transcription factor, MADS-box	A0A251VPA1
Helianthus annuus	MIKC MADS-box	HannXRQ_Chr02g0040221	HannXRQ_Chr02g0040221	Putative transcription factor, MADS-box, Transcription factor, K-box	A0A251VFG5
Helianthus annuus	MIKC MADS-box	HannXRQ_Chr02g0042981	CMB1	Putative MADS-box protein CMB1	A0A251VF33
Helianthus annuus	MIKC MADS-box	HannXRQ_Chr02g0043891	HannXRQ_Chr02g0043891	Putative transcription factor, MADS-box, Transcription factor, K-box	A0A251VFB5
Helianthus annuus	MIKC MADS-box	HannXRQ_Chr02g0045511	MADS2	Putative floral homeotic protein PMADS 2	A0A251VI85
Helianthus annuus	MADS-box	HannXRQ_Chr02g0058811	HannXRQ_Chr02g0058811	Putative MADS-box transcription factor family protein	A0A251VJZ6
Helianthus annuus	MADS-box	HannXRQ_Chr03g0074101	HannXRQ_Chr03g0074101	Putative transcription factor, MADS-box	A0A251V794

Helianthus annuus	MADS-box	HannXRQ_Chr03g0091451	AGL104	Putative agamous-like protein	A0A251VB91
Helianthus annuus	MIKC MADS-box	HannXRQ_Chr04g0110101	AGL8b	Putative agamous-like protein	A0A251UYX3
Helianthus annuus	MADS-box	HannXRQ_Chr04g0111861	AGL64	Putative AGAMOUS-like 64	A0A251V1L1
Helianthus annuus	MIKC MADS-box	HannXRQ_Chr04g0122131	AGL20	Putative AGAMOUS-like 20	A0A251V1D9
Helianthus annuus	MIKC MADS-box	HannXRQ_Chr05g0147261	HannXRQ_Chr05g0147261	Putative transcription factor, MADS-box, Transcription factor, K-box	A0A251URL9
Helianthus annuus	MIKC MADS-box	HannXRQ_Chr05g0149341	DEF21	Putative MADS-box protein defh21	A0A251UQI0
Helianthus annuus	MADS-box	HannXRQ_Chr05g0150571	HannXRQ_Chr05g0150571	Putative transcription factor, MADS-box	A0A251URQ0
Helianthus annuus	MIKC MADS-box	HannXRQ_Chr05g0150661	HannXRQ_Chr05g0150661	Putative transcription factor, MADS-box, Transcription factor, K-box	A0A251USH9
Helianthus annuus	MIKC MADS-box	HannXRQ_Chr05g0151331	MADS1	Putative floral homeotic protein PMADS 1	A0A251USE1
Helianthus annuus	MIKC MADS-box	HannXRQ_Chr05g0154261	HannXRQ_Chr05g0154261	Putative transcription factor, MADS-box, Transcription factor, K-box	A0A251URN3
Helianthus annuus	MADS-box	HannXRQ_Chr05g0156101	HannXRQ_Chr05g0156101	Putative transcription factor, MADS-box	A0A251US47
Helianthus annuus	MADS-box	HannXRQ_Chr05g0161331	HannXRQ_Chr05g0161331	Putative transcription factor, MADS-box	A0A251UUF4
Helianthus annuus	MIKC MADS-box	HannXRQ_Chr05g0163381	HannXRQ_Chr05g0163381	Putative transcription factor, MADS-box, Transcription factor, K-box	A0A251UUT7
Helianthus annuus	MIKC MADS-box	HannXRQ_Chr05g0163521	MAD27	Putative MADS-box transcription factor 27	A0A251UVI9
Helianthus annuus	MIKC MADS-box	HannXRQ_Chr06g0169281	HannXRQ_Chr06g0169281	Putative transcription factor, MADS-box, Transcription factor, K-box	A0A251UGL0
Helianthus annuus	MIKC MADS-box	HannXRQ_Chr06g0174721	AGL9b	Putative agamous-like protein	A0A251UIW6
Helianthus annuus	MIKC MADS-box	HannXRQ_Chr07g0200001	HannXRQ_Chr07g0200001	Putative transcription factor, MADS-box, Transcription factor, K-box	A0A251UCK2
Helianthus annuus	MIKC MADS-box	HannXRQ_Chr07g0200321	AGL9a	Putative agamous-like protein	A0A251UCK8
Helianthus annuus	MADS-box	HannXRQ_Chr07g0203621	HannXRQ_Chr07g0203621	Putative transcription factor, MADS-box	A0A251UDE8
Helianthus annuus	MIKC MADS-box	HannXRQ_Chr07g0203811	HannXRQ_Chr07g0203811	Putative transcription factor, MADS-box	A0A251UDH3
Helianthus annuus	MADS-box	HannXRQ_Chr08g0222641	HannXRQ_Chr08g0222641	Putative transcription factor, MADS-box	A0A251U4Y4
Helianthus annuus	MADS-box	HannXRQ_Chr08g0222651	HannXRQ_Chr08g0222651	Putative transcription factor, MADS-box	A0A251U6C4
Helianthus annuus	MIKC MADS-box	HannXRQ_Chr08g0223671	SEP4	Putative transcription factor, MADS-box, Transcription factor, K-box	A0A251U571
Helianthus annuus	MIKC MADS-box	HannXRQ_Chr08g0223681	HAM75	MADS-box transcription factor HAM75	Q8RVR0
Helianthus annuus	MADS-box	HannXRQ_Chr08g0229361	HannXRQ_Chr08g0229361	Putative transcription factor, MADS-box	A0A251U820
Helianthus annuus	MADS-box	HannXRQ_Chr08g0229541	HannXRQ_Chr08g0229541	Putative transcription factor, MADS-box	A0A251U7M1
Helianthus annuus	MADS-box	HannXRQ_Chr09g0258351	HannXRQ_Chr09g0258351	Putative transcription factor, MADS-box	A0A251TVT3
Helianthus annuus	MIKC MADS-box	HannXRQ_Chr09g0262151	HannXRQ_Chr09g0262151	Putative transcription factor, MADS-box, Transcription factor, K-box	A0A251TXU2

Helianthus annuus	MIKC MADS-box	HannXRQ_Chr09g0263231	HannXRQ_Chr09g0263231	Putative transcription factor, MADS-box	A0A251TYP2
Helianthus annuus	MIKC MADS-box	HannXRQ_Chr09g0265001	SEP1	Putative K-box region and MADS-box transcription factor family protein	A0A251TXJ6
Helianthus annuus	MIKC MADS-box	HannXRQ_Chr09g0266391	STK	Putative K-box region and MADS-box transcription factor family protein	A0A251TXW9
Helianthus annuus	MIKC MADS-box	HannXRQ_Chr09g0267081	AGL12	Putative AGAMOUS-like 12	A0A251TZ95
Helianthus annuus	MIKC MADS-box	HannXRQ_Chr09g0269421	HannXRQ_Chr09g0269421	Putative transcription factor, MADS-box, Transcription factor, K-box	A0A251TZS6
Helianthus annuus	MIKC MADS-box	HannXRQ_Chr09g0270311	HannXRQ_Chr09g0270311	Putative transcription factor, MADS-box, Transcription factor, K-box	A0A251TYW1
Helianthus annuus	MIKC MADS-box	HannXRQ_Chr09g0270841	HannXRQ_Chr09g0270841	Putative transcription factor, MADS-box, Transcription factor, K-box	A0A251TZ10
Helianthus annuus	MIKC MADS-box	HannXRQ_Chr09g0273361	CAL	Putative transcription factor CAULIFLOWER	A0A251U0N0
Helianthus annuus	MIKC MADS-box	HannXRQ_Chr10g0277721	HannXRQ_Chr10g0277721	Putative transcription factor, MADS-box	A0A251TFZ2
Helianthus annuus	MIKC MADS-box	HannXRQ_Chr10g0291191	ANR1	Putative AGAMOUS-like 44	A0A251THZ7
Helianthus annuus	MADS-box	HannXRQ_Chr10g0294541	HannXRQ_Chr10g0294541	Putative transcription factor, MADS-box	A0A251TKB4
Helianthus annuus	MADS-box	HannXRQ_Chr10g0296351	HannXRQ_Chr10g0296351	Putative transcription factor, MADS-box	A0A251TK21
Helianthus annuus	MADS-box	HannXRQ_Chr10g0296361	HannXRQ_Chr10g0296361	Putative MADS-box transcription factor family protein	A0A251TJB2
Helianthus annuus	MIKC MADS-box	HannXRQ_Chr10g0299591	HannXRQ_Chr10g0299591	Putative transcription factor, MADS-box, Transcription factor, K-box	A0A251TL78
Helianthus annuus	MADS-box	HannXRQ_Chr11g0322011	HannXRQ_Chr11g0322011	Putative transcription factor, MADS-box	A0A251T6F5
Helianthus annuus	MADS-box	HannXRQ_Chr11g0322021	HannXRQ_Chr11g0322021	Putative transcription factor, MADS-box	A0A251T824
Helianthus annuus	MADS-box	HannXRQ_Chr11g0350681	HannXRQ_Chr11g0350681	Putative transcription factor, MADS-box	A0A251TEC6
Helianthus annuus	MIKC MADS-box	HannXRQ_Chr12g0358711	MADS6	Putative MADS-box transcription factor 6	A0A251T0C1
Helianthus annuus	MIKC MADS-box	HannXRQ_Chr12g0358721	AGL42	Putative AGAMOUS-like 42	A0A251SZX1
Helianthus annuus	MIKC MADS-box	HannXRQ_Chr12g0358781	PI	Putative K-box region and MADS-box transcription factor family protein	A0A251SZZ4
Helianthus annuus	MIKC MADS-box	HannXRQ_Chr12g0361321	MTF1	Putative MADS-box transcription factor 1	A0A251T0J0
Helianthus annuus	MIKC MADS-box	HannXRQ_Chr12g0364981	HAM92	MADS-box transcriptional factor HAM92	Q84LC0
Helianthus annuus	MIKC MADS-box	HannXRQ_Chr12g0375471	MAF1	Putative K-box region and MADS-box transcription factor family protein	A0A251T3A9
Helianthus annuus	MIKC MADS-box	HannXRQ_Chr12g0375571	HannXRQ_Chr12g0375571	Putative transcription factor, MADS-box	A0A251T3C0
Helianthus annuus	MIKC MADS-box	HannXRQ_Chr13g0405061	JOIN	Putative MADS-box protein JOINTLESS	A0A251SSA2
Helianthus annuus	MADS-box	HannXRQ_Chr14g0431471	AGL23	Putative AGAMOUS-like 23	A0A251SFV6
Helianthus annuus	MADS-box	HannXRQ_Chr14g0431651	AGL28	Putative AGAMOUS-like 28	A0A251SEV4

Helianthus annuus	MADS-box	HannXRQ_Chr14g0431671	HannXRQ_Chr14g0431671	Putative transcription factor, MADS-box	A0A251SE86
Helianthus annuus	MIKC MADS-box	HannXRQ_Chr14g0452461	AGL8a	Putative agamous-like protein	A0A251SKI4
Helianthus annuus	MADS-box	HannXRQ_Chr14g0456481	AGL61	Putative AGAMOUS-like 61	A0A251SKS0
Helianthus annuus	MADS-box	HannXRQ_Chr14g0456491	HannXRQ_Chr14g0456491	Putative transcription factor, MADS-box	A0A251SLC6
Helianthus annuus	MADS-box	HannXRQ_Chr15g0464231	HannXRQ_Chr15g0464231	Putative transcription factor, MADS-box	A0A251S4B4
Helianthus annuus	MIKC MADS-box	HannXRQ_Chr15g0474011	MAD23	Putative MADS-box transcription factor 23	A0A251S7Q3
Helianthus annuus	MADS-box	HannXRQ_Chr15g0479251	HannXRQ_Chr15g0479251	Putative transcription factor, MADS-box	A0A251S8M6
Helianthus annuus	MADS-box	HannXRQ_Chr15g0479261	HannXRQ_Chr15g0479261	Putative transcription factor, MADS-box	A0A251S8E4
Helianthus annuus	MADS-box	HannXRQ_Chr15g0479291	HannXRQ_Chr15g0479291	Putative transcription factor, MADS-box	A0A251SA06
Helianthus annuus	MADS-box	HannXRQ_Chr15g0479301	HannXRQ_Chr15g0479301	Putative transcription factor, MADS-box	A0A251S8B8
Helianthus annuus	MADS-box	HannXRQ_Chr15g0479321	HannXRQ_Chr15g0479321	Putative transcription factor, MADS-box	A0A251S8X4
Helianthus annuus	MADS-box	HannXRQ_Chr15g0483111	HannXRQ_Chr15g0483111	Putative transcription factor, MADS-box	A0A251S987
Helianthus annuus	MIKC MADS-box	HannXRQ_Chr15g0484901	AGL24	Putative AGAMOUS-like 24	A0A251SDU1
Helianthus annuus	MIKC MADS-box	HannXRQ_Chr15g0489071	HannXRQ_Chr15g0489071	Putative transcription factor, MADS-box, Transcription factor, K-box	A0A251SB60
Helianthus annuus	MIKC MADS-box	HannXRQ_Chr15g0495861	HannXRQ_Chr15g0495861	Putative transcription factor, MADS-box, Transcription factor, K-box	A0A251SE94
Helianthus annuus	MIKC MADS-box	HannXRQ_Chr16g0501381	HannXRQ_Chr16g0501381	Putative transcription factor, MADS-box, Transcription factor, K-box	A0A251S0B0
Helianthus annuus	MIKC MADS-box	HannXRQ_Chr16g0503081	HannXRQ_Chr16g0503081	Putative transcription factor, MADS-box, Transcription factor, K-box	A0A251RWB8
Helianthus annuus	MIKC MADS-box	HannXRQ_Chr16g0507561	HannXRQ_Chr16g0507561	Putative transcription factor, MADS-box, Transcription factor, K-box	A0A251RXF9
Helianthus annuus	MIKC MADS-box	HannXRQ_Chr16g0519321	AG	Putative floral homeotic protein AGAMOUS	A0A251S0H6
Helianthus annuus	MIKC MADS-box	HannXRQ_Chr16g0523531	ALG14	Putative AGAMOUS-like 14	A0A251S1N8
Helianthus annuus	MIKC MADS-box	HannXRQ_Chr16g0530971	MAD16	Putative MADS-box transcription factor 16	A0A251S7R8
Helianthus annuus	MIKC MADS-box	HannXRQ_Chr17g0538691	MAF4	Putative K-box region and MADS-box transcription factor family protein	A0A251RMB7
Helianthus annuus	MIKC MADS-box	HannXRQ_Chr17g0550551	AGL31	Putative AGAMOUS-like 31	A0A251RPX1
Helianthus annuus	MIKC MADS-box	HannXRQ_Chr17g0560051	HannXRQ_Chr17g0560051	Putative transcription factor, MADS-box, Transcription factor, K-box	A0A251RSS6
Helianthus	FT	HannXRQ_Chr01g0024111	HannXRQ_Chr01g0024111	Putative phosphatidylethanolamine-binding protein PEBP	A0A251VQQ7
Helianthus	FT	HannXRQ_Chr06g0183471	HannXRQ_Chr06g0183471	Putative phosphatidylethanolamine-binding protein PEBP	A0A251UKE2
Helianthus	FT	HannXRQ_Chr06g0183481	HannXRQ_Chr06g0183481	Putative phosphatidylethanolamine-binding protein PEBP	A0A251UK94
Helianthus	FT	HannXRQ_Chr07g0197921	TFL1	Putative CEN-like protein 2	E3TQ07
Helianthus	FT	HannXRQ_Chr07g0206891	HannXRQ_Chr07g0206891	Putative phosphatidylethanolamine-binding protein PEBP	A0A251UFX8
Helianthus	FT	HannXRQ_Chr07g0207081	HannXRQ_Chr07g0207081	Putative phosphatidylethanolamine-binding protein PEBP	A0A251UED9

Helianthus	FT	HannXRQ_Chr08 g0220851	HannXRQ_Chr 08g0220851	Putative phosphatidylethanolamine-binding protein PEBP	A0A251 U629
Helianthus	FT	HannXRQ_Chr08 g0220861	HannXRQ_Chr 08g0220861	Putative phosphatidylethanolamine-binding protein PEBP	A0A251 U5A7
Helianthus	FT	HannXRQ_Chr08 g0234381	FT4	Flowering locus T4	D5I5U9
Helianthus	FT	HannXRQ_Chr11 g0351631	HannXRQ_Chr 11g0351631	Putative phosphatidylethanolamine-binding protein PEBP	A0A251 TDT7
Helianthus	FT	HannXRQ_Chr13 g0393741	CET1	Putative CEN-like protein 1	A0A251 SPZ8
Helianthus	FT	HannXRQ_Chr13 g0393751	HannXRQ_Chr 13g0393751	Putative phosphatidylethanolamine-binding protein PEBP	A0A251 SPB2
Helianthus	FT	HannXRQ_Chr13 g0399101	HannXRQ_Chr 13g0399101	Putative phosphatidylethanolamine-binding protein PEBP	A0A251 SSA8
Helianthus	FT	HannXRQ_Chr14 g0462261	HannXRQ_Chr 14g0462261	Putative phosphatidylethanolamine-binding protein PEBP	A0A251 SMW3
Arabidopsis	FT	At1g18100	MFT	Protein MOTHER of FT and TFL1	Q9XFK7
Arabidopsis	FT	At1g65480	FT	Protein FLOWERING LOCUS T	Q9SXZ2
Arabidopsis	FT	At2g27550	CEN	Protein CENTRORADIALIS-like	Q9ZNV5
Arabidopsis	FT	At4g20370	TSF	Protein TWIN SISTER of FT	Q9S7R5
Arabidopsis	FT	At5g03840	TFL1	Protein TERMINAL FLOWER 1	P93003
Arabidopsis	FT	At5g62040	BFT	Protein BROTHER of FT and TFL 1	Q9FIT4
